# Competing Voices, Persistent Gaps: Shareholder Proposal Activism and ESG Decoupling

**DOI:** 10.1177/00076503251357372

**Published:** 2025-08-20

**Authors:** Eduardo Schiehll, Henrique Castro Martins, Krista B. Lewellyn

**Affiliations:** 1 HEC-Montréal, QC, Canada; 2 Fundação Getúlio Vargas—FGV/EAESP, São Paulo, Brazil; 3 Florida Southern College, Lakeland, USA

**Keywords:** competing demands, ESG decoupling, intra-institutional complexity, investor orientation, shareholder activism

## Abstract

We investigate the occurrence and the persistence of environmental, social, and governance (ESG) decoupling when firms are exposed to heterogeneity in shareholder proposal activism, reflected by the different orientation of shareholder activists and the competing nature of their demands. We find that the greater the extent that shareholder proposals blend financial and social logics, the more likely firms are to respond by adopting policies that are not fully implemented in practice. Our results also suggest that decoupling is more persistent among targeted firms that are exposed to heightened heterogeneity in shareholder proposal activism. Our findings suggest that such heterogeneity not only prompts decoupling but also leads to its persistence, contributing to our understanding about why firms may place their legitimacy at risk to respond to shareholder activism, and the conditions that motivate (or inhibit) such behavior. Our study contributes to the symbolic management literature and the ongoing debate on whether and how shareholders impact societal challenges.

Driven by the rise of environmental, social, and governance (ESG) concerns, a growing consensus is emerging that firms should balance financial and social goals. This consensus, however, is shaped by competing and often conflicting financial and social logics, reflected in the diverse expectations of multiple stakeholders. In this situation, firms often respond in symbolic ways by decoupling their stated policies from actual practices to avoid punishment and retain support in the eyes of stakeholders (Bromley & Powell, 2012; Graafland & Smid, 2019; J. W. Meyer & Rowan, 1977; Westphal & Zajac, 2001). Yet these stakeholders are often conceptualized as homogeneous groups, even though growing evidence reveals important intra-group variation (Briscoe & Gupta, 2016), particularly among shareholders (Yan et al., 2021) whose orientations and demands may diverge sharply. This intra-group heterogeneity complicates firms’ efforts to respond effectively, as the competing demands arise and are enforced within the same stakeholder group and thereby cannot be addressed through conventional audience segmentation or tailored responses (R. E. Meyer & Höllerer, 2016, Yan, 2020).

To examine this underexplored context where competing expectations associated with financial and social logics are endorsed by the same constituency, we focus on shareholder activism, which is expressed through shareholder-initiated proposals and involves diverse demands from shareholder activists with different orientations (Chuah et al., 2024; DesJardine et al., 2023). In contrast to many other stakeholder groups, “*shareholders occupy a privileged position to place formal demands on companies and their executives*” (DesJardine et al., 2023, p. 401). The heightened influence over managers is due to shareholders having economic power and legal rights that comes from their ownership positions (Vasi & King, 2012). Shareholders, as stakeholders with a residual claim to the firm’s value, can use their position to express divergent interests and exert pressure through both formal and informal mechanisms.

Contrasting with conventional depictions of shareholders being driven by a singular profit-oriented logic, recent research demonstrates significant heterogeneity among shareholders in terms of their characteristics and priorities (DesJardine et al., 2024). For instance, some shareholders adhere predominantly to socially responsible investing (SRI), advocating for firms to pursue a social performance logic alongside the conventional profit-oriented logic (DesJardine et al., 2023; Gibson et al., 2022; Gond & Piani, 2013). Indeed, socially oriented shareholder activism (SOSA) “*has increased in frequency, legitimacy and institutional acceptance*” (Hadani et al., 2018, p. 2065) and challenges the central financial logic of investment practices (Yan et al., 2019). To illustrate this phenomenon, consider the 20 shareholder-initiated proposals appearing on the 2020 Amazon Proxy Ballot, 17 of which can be considered as socially oriented, since they challenge management to take action on social-related issues such as human rights, employee diversity, individuals’ data security, and corporate political activity. Surprisingly, among the 17 proposals, almost half (8) of these proposals were initiated by shareholder activists that are not classified as predominantly adhering to SRI (i.e., non-SRI activists).

Shareholder proposal activism places pressures on targeted firms by amplifying competing legitimate logics within the same institutional order (R. E. Meyer & Höllerer, 2016; Yan et al., 2021). Specifically, shareholder activists with differing identities and competing demands, oriented along a continuum between financial and social logics, generate what is referred to in the literature as *intra-institutional complexity* (R. E. Meyer & Höllerer, 2016; Yan, 2020; Yan et al., 2021). While previous research has focused on inter-institutional complexity, assuming that competing logics arise only from different and independent stakeholder groups (e.g., environmental activists vs. labor groups), intra-institutional complexity occurs when divergent logics emerge within the same stakeholder group, such as shareholders committed to SRI and conventional, financially focused shareholders (Yan, 2020; Yan et al., 2021).

Further, in contrast with other forms of stakeholder activism (e.g., boycotts, strikes, protests) shareholder proposal activism is a setting with an acute degree of ambiguity, because competing demands are enforced and highly coordinated by the same constituency (Greenwood et al., 2011), through the more coercive and formal mechanisms that exist for investor protection (Bates & Hennessy, 2010). Prior research has largely examined the effects of shareholder activists’ strategies, interests, and demands in two main ways: by focusing independently on a particular type of activism—socially or non-socially oriented—or by applying a broad treatment to shareholder activism—where different activist identities and demands are aggregated and treated as equivalent. These approaches overlook “*the heterogenous origins of activism between different shareholder types*” (Chuah et al., 2024, p. 8), which expose targeted firms to competing demands grounded in different institutional logics (Ioannou & Serafeim, 2015) endorsed simultaneously by the same stakeholder group (i.e., shareholders) (Sikavica et al., 2020). As Chuah et al. (2024) suggest, SOSA differs from the more traditional activism on governance or financial performance, and, while now fully institutionalized, often triggers greater opposition due to its challenge to established corporate priorities.

We contend that intra-institutional complexity arising from heterogeneous shareholder proposal activism contributes to the occurrence and persistence of ESG policy-practice decoupling for two main reasons. First, SOSA reflects shareholders’ formal disapproval of the misalignment between firm behavior and societal expectations, highlighting that environmental and social externalities are no longer seen as peripheral but as salient to shareholders’ decision-making. In contrast to governance and financial issues (e.g., voting rights, board composition, dividend, or assets reallocation), addressing environmental and social externalities that are increasingly perceived as salient by the shareholder base may require significant structural changes on the part of the firm. Thus, the need to pacify different shareholder activists and avoid further scrutiny may lead to gaps between claimed policies and effective implementation. Second, when shareholder activism is characterized by heterogeneity in shareholder activists’ identities and demands, ESG decoupling tends to persist, as firms face greater difficulty reconciling competing demands than when shareholder activism is grounded in a single, coherent logic (Greenwood et al., 2011). Firms often decouple practice from policy to evade or manage scrutiny. However, when this scrutiny originates from shareholder activism grounded in a common unified logic, such as a shared financial orientation, firms face more coherent and clearly defined demands, which limits the persistence of ESG decoupling (Boxenbaum & Jonsson, 2017). In contrast, heterogeneity in shareholder identity and demands introduces competing logics with less-defined boundaries (Chuah et al., 2024; Sikavica et al., 2020), making it harder for firms to reconcile shareholder expectations and thereby policy-practice decoupling is more persistent.

To test our hypotheses about the influence of shareholder proposal activism heterogeneity on the occurrence and persistence of ESG police-practice decoupling, we draw upon a sample of shareholder-initiated proposals submitted to S&P 1500 firms between 2013 and 2021. While acknowledging that shareholder activism can manifest in a variety of ways (Denes et al., 2017), we focus on shareholder proposal activism for several reasons. Unlike more costly proxy battles, shareholder-initiated proposals offer a low-cost avenue for shareholder engagement, which allows even minority or individual shareholders to publicly voice their expectations (Benton & You, 2019). Relatedly, submitting proposals does not require private access or personal contact with company management, thus making it a tactic more readily accessible to a wider range of shareholders, such as special interest groups, labor unions, or individual shareholders. In addition, institutional and large investors tend to sponsor proposals if and when their private negotiations with management become ineffective (Clark et al., 2017). Finally, unlike informal tactics, shareholder proposals are publicly disclosed, such that they can lead to media coverage, raising reputational concerns for firm executives.^1^ Our sample, therefore, comprises 6,446 shareholder-initiated proposals targeting 1,506 nonfinancial firms from 2013 to 2021, for which we collected scores for ESG reporting and ESG performance from the same rating agency (S&P Global) to capture the degree of ESG policy-practice decoupling (Graafland & Smid, 2019; Sauerwald & Su, 2019; Tashman et al., 2019). To measure the heterogeneity in shareholder proposal activism, we classified proposals relative to the identity of the shareholders sponsoring the proposals (SRI or non-SRI) as well as the type of demand put forward by these shareholders (SOSA or non-SOSA). We find that when shareholder proposal activism blends financial and social logics to a greater extent (i.e., greater heterogeneity), firms are more likely to respond with symbolic actions, for a consequential misalignment between advocated ESG policies and implemented ESG practices. Our results also demonstrate that ESG policy-practice decoupling is more persistent in targeted firms that experience more heterogeneous shareholder proposal activism. Our results are robust to alternative measures of ESG policy-practice decoupling and different model specifications.

With this research, we seek to contribute to theory and practice in several ways. First, we explore shareholder proposal activism as a source of intra-institutional complexity that facilitates and sustains policy-practice decoupling, contributing to domains associated with symbolic organizational responses to competing demands (Greenwood et al., 2011). While prior research has highlighted that conflicting expectations from different stakeholder groups often lead firms to engage in decoupling (Crilly et al., 2012; Testa et al., 2018), it remains unclear how firms adapt and respond to competing demands from the same but heterogenous stakeholder group (i.e., shareholders) (Boxenbaum & Jonsson, 2017; Hawn & Ioannou, 2016). Additionally, prior literature has primarily examined the effects of shareholder activism either on firm performance or on disclosure outcomes in isolation, leaving an important question unanswered: Are firms’ responses to shareholder activism symbolic or substantial? This oversight limits our understanding of firms’ responses to shareholder activists with different investment orientations and competing demands, which are oriented along a continuum between financial and social logics. By examining this underexplored context from an intra-institutional complexity perspective, our study extends the shareholder activism literature and responds to recent calls for research that addresses “*how do firms adapt to heterogenous and conflicting demands by shareholder activists?*” (Chuah et al., 2024, p. 65).

Our conceptualization of the heterogeneity in shareholder activism as a source of intra-institutional complexity constitutes a new way of looking at the impact shareholder activism has on organizational outcomes, specifically with regard to symbolic responses. Further, our findings on the persistence of decoupling shed light on why firms might jeopardize their legitimacy for extended periods in response to shareholder activism. Finally, by disaggregating heterogeneity of shareholder proposal activism along the dimensions of shareholder identity and type of demands, we reveal important nuances concerning the ongoing debate on the impact shareholders may have on societal challenges.

## Theory and Hypotheses

Firms are confronted with institutional complexity when there are incompatible demands that dictate what constitutes suitable corporate conduct (Greenwood et al., 2011). When demands are incompatible, they generate competing expectations about appropriate organizational goals and create uncertainty about which goals should prevail. By contrast, when demands are compatible, they reinforce consistent expectations and reduce ambiguity in organizational decision-making (Ioannou & Serafeim, 2015; Pache & Santos, 2013). Policy-practice decoupling refers to the process whereby a firm adopts a formal policy to gain legitimacy without completely implementing it in daily practices (Bromley & Powell, 2012; Graafland & Smid, 2017; Haack & Schoeneborn, 2015).

To better understand why firms may respond in substantial or symbolic ways to such complexity, Greenwood et al. (2011) suggest considering the degree of fragmentation and formalization of competing demands, and how these attributes strengthen (or weaken) the pressure of competing logics on firms. A highly fragmented context is one in which competing logics are associated with different and independent stakeholder groups, allowing firms to segment their audience when responding to their demands, “*demands that are, at best, uncoordinated*” (Greenwood et al., 2011, p. 320). While formalization refers to the degree to which these competing logics emerge from more organized groups of constituents (Greenwood et al., 2011; J. W. Meyer & Rowan, 1977; Pache & Santos, 2013). Shareholder-initiated proposals reflect institutionalized and coercive power via ownership position, public disclosure, and formal shareholder voting rights (Bates & Hennessy, 2010). Accordingly, shareholder proposals represent public and organized actions (Michelon et al., 2020) by a residual claimant stakeholder group with low resource dependence on the targeted firm (Briscoe & Gupta, 2016). Hence, when competing demands are enforced and coordinated by the same powerful constituency, as in the case of shareholder activism, firms face a context of acute incompatibility. In such situations, separating audiences and value orientations—based on whether social or financial goals are prioritized—is less feasible (Yan et al., 2019), reflecting “*intra-institutional complexity*” (R. E. Meyer & Höllerer, 2016, p. 374).

Shareholders who give importance to environmental, social, and ethical issues when making investment decisions (i.e., SRIs), adhere predominantly to social logics (DesJardine et al., 2023; Yan et al., 2019, 2021). SRIs have become increasingly salient activists alongside their purely profit-oriented counterparts (non-SRIs) (Gond & Piani, 2013). This implies that expectations within a firm’s shareholder base can be disparate and enhance the financial/social trade-offs that a targeted firm can experience, because social goals are perceived to belong to a distinct logic and contradict the premises of financial logic in investment practice (Yan, 2020; Yan et al., 2019). Along with this difference in shareholder activists’ adherence to social goals (i.e., SRI; non-SRI), prior literature has documented the prevalence of heterogeneity in shareholder activists’ demands (Clark et al., 2017; Goranova & Ryan, 2014; Renneboog & Szilagyi, 2011). Arising from the social justice movement, SOSA seeks to enact improvements in firms’ policies and practices (e.g., diversity, pay gap, product, and labor safety) for the benefit of a wider group of stakeholders. In contrast, the goal of non-SOSA is assumed to be capital accumulation, to improving corporate governance and financial performance, predominantly for the benefit of shareholders (Renneboog & Szilagyi, 2011).

Accordingly, we maintain that profit-oriented and social performance logics co-exist in shareholder proposal activism and are reflected by both the differences in the adherence to social goals of shareholders sponsoring these proposals (i.e., SRI or non-SRI) and the nature of their demands (i.e., SOSA or non-SOSA). As such, shareholder proposal activism offers a setting where competing demands are fragmented but formalized and well-coordinated by the same constituency. Fragmentation occurs because shareholder activism involves competing demands (SOSA and non-SOSA) sponsored by shareholders with different orientations (SRI and non-SRI). Moreover, shareholder proposals are well-coordinated, visible, and institutionalized through the mechanisms for investor protection (Bates & Hennessy, 2010).^2^ Therefore, we focus on the heterogeneity in shareholder proposals, both in terms of the variety of underlying logics they incorporate (SOSA and non-SOSA) and the diverse identities of the shareholder activists who sponsor these proposals (SRI and non-SRI) (R. E. Meyer & Höllerer, 2016; Yan, 2020; Yan et al., 2019).

### Shareholder Proposal Activism and the Occurrence of Decoupling

Extant literature shows that shareholders differ considerably in terms of class, characteristics, and the tactics they use to influence firms they invest in (Clark et al., 2017; Sikavica et al., 2020). Recently, DesJardine et al. (2023) identified investment orientation as an important dimension for understanding the extent to which shareholders’ interests converge or diverge with the interests of non-equity stakeholders.^3^ Investment orientation can be viewed as a continuum, with one extreme represented by shareholders focused solely on maximizing financial gains (i.e., non-SRI), and at the opposite end are shareholders whose investment intentions consider long-term impact on other stakeholders (i.e., SRI) by focusing on social performance, not just financial gains (DesJardine et al., 2023). Along this continuum, shareholders’ relative commitment to achieving financial and social goals through their investments differ (Yan et al., 2019) based on the type of value they attempt to create, and their fiduciary responsibilities (Luo et al., 2017; Sikavica et al., 2020). For example, union pension funds may be more concerned with employee welfare than with the stock price of targeted firms. Mutual funds tend to focus primarily on governance-based financial activism, seeking to improve governance structures and make managers more accountable to shareholders (Goranova & Ryan, 2014).

With respect to the nature of activists’ demands, profit- and governance-oriented activism (i.e., non-SOSA), arises when firm financial performance diverges from shareholders’ expectations (Denes et al., 2017). In contrast, SOSA tends to occur when investors observe that the firm is moving away from current industry or firm-level practices with respect to what is ethically or socially acceptable, prompting a change in shareholders’ risk perceptions (Hadani et al., 2018; Vasi & King, 2012). SOSA, therefore, can be seen as a public signal of shareholder concerns about the focal firm’s environmental and social externalities, drawing management’s attention to the focal ESG issue, as they are compelled to mitigate and disclose information on this externality to satisfy investors’ expectations. Additionally, signifying an environmental or social issue as salient in the eyes of shareholders (Ioannou & Serafeim, 2015) requires management to allocate incremental resources to address the issue, as well as internal controls to monitor the externality (Hawn & Ioannou, 2016). Further, and perhaps most importantly, it elevates the focal ESG issue to one for consideration, diligence, and integration into the firm’s governance processes and risk management systems.

Building on the above, we propose that the combination of shareholder activists with different relative adherence to social goals (SRI and non-SRI) and the different nature of demands advocated in their proposals (i.e., SOSA vs. non-SOSA; Goranova & Ryan, 2014; Hadani et al., 2018; Sikavica et al., 2020) reflects the degree of trade-offs between profit-oriented and social performance that a targeted firm is exposed to. Consequently, greater heterogeneity in shareholder proposal activism increases the need for targeted firms to reconcile the competing demands. Stated formally:

**Hypothesis 1:** Heterogeneity in shareholder proposal activism is positively associated with ESG policy-practice decoupling.

### Shareholder Proposal Activism and the Persistence of Decoupling

Another important question is whether policy-practice decoupling persists over time. As Bromley and Powell (2012) suggest, when externally driven demands conflict with a firm’s dominant financial logic, a rational response is to avoid or delay implementation, but as rationalization proceeds, the alignment of policy and practice (i.e., recoupling) becomes a moral obligation. Although previous research suggests that stakeholders may not necessarily penalize firms that exhibit evidence of decoupling, in the long run, decoupling may backfire, causing a loss of stakeholder support and firm legitimacy (Boxenbaum & Jonsson, 2017; García-Sánchez et al., 2021). Accordingly, the amount of time that a firm can “talk the talk” but not “walk the walk” may be limited because stakeholder groups representing well-specified logic domains will exert pressure for corrective actions and over time, firms may have no choice but to recouple policies with performance (Boxenbaum & Jonsson, 2017).

More precisely, prior research suggests that when firms face competing demands from distinct stakeholder groups (i.e., inter-institutional complexity), the separation of expectations across constituencies can facilitate clearer evaluation and oversight (Briscoe & Gupta, 2016), constraining the persistence of policy-practice decoupling. In contrast, in the context of shareholder proposal activism, where competing demands are rooted in varying degrees of emphasis on financial versus social goals (i.e., SOSA non-SOSA) and channeled through a single, powerful constituency with different orientations (SRI, non-SRI), firms cannot easily compartmentalize or sequence their responses to different shareholder expectations. Hence, intra-institutional complexity arises because “*splitting the audience or attending to them in turns is not an option*” (R. E. Meyer & Höllerer, 2016, p. 375). As a result, expectations become ambiguous over time, limiting shareholders’ ability to exercise oversight and to determine whether firm actions are symbolic or substantive. In this context, there are reasons to expect that heightened heterogeneity in shareholder proposal activism will lead to more persistent decoupling than when shareholder proposal activism reflects a coherent logic (i.e., homogenous proposals).

In addition, with the rise of social justice movements, SRI activists have used their ownership position and voting rights to emphasize the importance of achieving environmental and social goals alongside financial performance (Ioannou & Serafeim, 2015). In this way, SRI activists are vying for time and attention with mainstream financial investment practices (Hadani et al., 2018; Yan et al., 2019). Thus, we contend that when shareholders simultaneously demand actions that serve both profit-oriented and social performance goals, the lack of well-defined boundaries complicates the process of oversight, making it harder to distinguish between symbolic and substantive actions (Luo et al., 2017), leading firms to persist in proclaiming commitment to ESG policies to address shareholders’ expectations, while delaying or symbolically implementing them. In other words, responding to SRI activists’ expectations may enhance firms’ legitimacy and social responsibility but may reduce resource gains and alignment with other shareholders-centric financial logics (Yan et al., 2019). In this heightened state of ambiguity, ESG policy-practice decoupling can become a persistent mechanism to accommodate shareholder activists and delay further scrutiny. Consequently, ESG policy-practice decoupling emerges as a persistent mechanism. Accordingly, we propose:

**Hypothesis 2:** Heterogeneity in shareholder proposal activism increases the persistence of ESG policy-practice decoupling.

## Methods

### Sample and Data

To construct our sample, we first identified all firms included in the S&P 1500 index during the period covered by our analysis (2013–2021).^4^ We then identified the availability of ESG scores for our sample firms from the *S&P Global ESG Scores* database, resulting in a target sample of 8,449 firm-year observations from 1,506 unique firms. The next step was to collect financial data from *Compustat*, institutional ownership data from *Refinitiv Global Ownership*, and the entrenchment index from the *ISS Governance Dataset*. We excluded firm-year observations with missing data from these sources, as well as firms belonging to the financial sector (2-digit SIC codes 60-67: Finance, Insurance, Real Estate) and the Fama & French industry named “Everything Else” (2-digit SIC codes: 49). We then searched all shareholder-initiated proposals that targeted our sample firms during the analysis period, as listed in the *Institutional Shareholder Services* (ISS) dataset on shareholder proxy resolutions. Our interest is not whether these proposals are voted on or implemented individually, but rather do they collectively “*bring important issues to the attention of the management*” (Flammer et al., 2021, p. 1861).^5^ We merged the data from all these sources using firms’ ticker symbols, and in some cases, we cross-checked by company names, CUSIP, ISIN codes, and ISS identifiers to ensure that all available data were correctly matched. The final sample comprises 5,577 firm-year observations, of which 3,457 were targets of shareholder proposal activism during the analysis period and were subject to a total of 6,446 shareholder-initiated proposals. Table 1 summarizes the sampling procedure.

**Table 1.** Sample Description 2013 to 2021.

Sample selection criteria	Full sample		
S&P 1500 firms with S&P Global ESG Performance and Reporting Score available.	8,449	1,506 firms	
(-) Missing data for financials, entrenchment, and ownership; excluding firms in the financial sector.	(2,872)		
Target sample (*Firm-year obs*)	5,577	Non-targeted	Targeted
Overlap with ISS proposal dataset (*Firm-year obs*)		2,120	3,457
Number of shareholder-initiated *proposals*			6,446

*Note*. ESG = environmental, social, and governance; ISS = *Institutional Shareholder Services*.

Table 2, Panel A, presents the industry distribution of shareholder-initiated proposals in our sample. The results show that shareholders proposals are concentrated in some industries, with Healthcare, Medical Equipment, Pharmaceutical Products (723, 11.22%), Petroleum and Natural Gas (672, 10.43%), Retail (667, 10.35%), and Utilities (641, 9.94%), accounting for more than 40% of all shareholders-initiated proposals in our sample period. When looking at the industry distribution by type of proposal, SOSA and non-SOSA, we observe that all industries receive both types of proposals.

**Table 2.** Description of Shareholder-Initiated Proposals for the Full Sample.

Panel A: Distribution of proposals by Fama and French’s (1997) 30 industry classification				
Industry name	SOSA	Non-SOSA	*N*	Percentage (%)
1. Food products	194	98	292	4.53
2. Beer and liquor	38	24	62	0.96
3. Tobacco products	44	7	51	0.79
4. Recreation	18	71	89	1.38
5. Printing and publishing	7	13	20	0.31
6. Consumer goods	47	52	99	1.54
7. Apparel	42	21	63	0.98
8. Healthcare, medical equipment, pharmaceutical products	291	432	723	11.22
9. Chemicals	52	88	140	2.17
10. Textiles	2	2	4	0.06
11. Construction and construction materials	34	72	106	1.64
12. Steel works, etc.	29	20	49	0.76
13. Fabricated products and machinery	65	152	217	3.37
14. Electrical equipment	5	9	14	0.22
15. Automobiles and trucks	38	103	141	2.19
16. Aircraft, ships, and railroad equipment	43	112	155	2.40
17. Precious metals, non-metallic, and industrial metal mining	18	22	40	0.62
18. Coal	12	5	17	0.26
19. Petroleum and natural gas	412	260	672	10.43
20. Utilities	396	245	641	9.94
21. Communication	158	196	354	5.49
22. Personal and business services	207	359	566	8.78
23. Business equipment	210	253	463	7.18
24. Business supplies and shipping containers	24	56	80	1.24
25. Transportation	149	178	327	5.07
26. Wholesale	42	94	136	2.11
27. Retail	387	280	667	10.35
28. Restaurants, hotels, motels	157	101	258	4.00
Total	3,121	3,325	6,446	100

Panel B: Distribution of proposals by activist identity (*N* = 5,723)^a^					
SRI activists			Non-SRI activists		
SRI type	Obs.	Percentage	Non-SRI type	Obs.	Percentage
Coordinated (SRI)	107	1.87	Corporate	133	2.32
Faith-based funds	507	8.86	Coordinated (non-SRI)	104	1.82
Individual shareholders	142	2.48	Hedge and mutual funds	352	6.15
SRI funds	787	13.75	Individual shareholders	1,460	25.51
Special interest groups	384	6.71	Other shareholder groups	626	10.94
Union pension funds	363	6.34	Public pension funds	758	13.24
Total	2,290	40.01	Total	3,433	59.99

Panel C: Distribution of proposals by type of demand (*N* = 6,446)							
SOSA				Non-SOSA			
Type	Code	Obs.	Percentage	Type	Code	Obs.	Percentage
Community	C	980	15.20	Auditor related	AUD	37	0.57
Diversity	D	54	0.84	Business operations	BO	12	0.19
Employee	ER	308	4.78	Board of directors	BOD	1,579	24.50
Human rights	HR	242	3.75	Codes, policy, standards	CPS	345	5.35
Product	P	364	5.65	Executive compensation	EXC	481	7.46
Emissions	GHG	550	8.53	Financing	FIN	33	0.51
Waste Mgmt.	WM	87	1.35	Governance	G	449	6.97
Water	W	25	0.39	Income allocation	INC	16	0.25
Environment—Misc.	EM	118	1.83	Transparency	TR	61	0.95
Sustainability Discl.	SD	393	6.10	Voting rights	VOTE	312	4.84
Total		3,121	48.42	Total		3,325	51.58

Panel D: Distribution of proposals by activist identity vs. proposals’ type of demands (*N* = 5,723)				
		Proposal type		Total
		Non-SOSA	SOSA	
Activist type	Non-SRI	2,334	1,099	3,433
	SRI	499	1,791	2,290
	Total	2,833	2,890	5,723

*Note*. ISS = *Institutional Shareholder Services*; SOSA = socially oriented shareholder activism; SRI = socially responsible investing.

a The difference in the number of proposals between Panels B and C is due to 236 proposals missing the “sponsor identity” classification in the ISS dataset and 487 proposals sponsored by individual shareholders that were not classified as either SRI or non-SRI investors.

### Dependent Variable

Consistent with previous studies (García-Sánchez et al., 2021; Graafland & Smid, 2019; Hawn & Ioannou, 2016; Sauerwald & Su, 2019; Tashman et al., 2019), we conceptualize firms’ ESG policy practice decoupling as the degree of misalignment between the information reported and the corresponding performance on the same set of ESG issues. The rationale for this measure is that ESG performance reflects a firm’s internal actions implemented to achieve structural changes and often involves adopting widely accepted ESG practices. In contrast, external actions generally focus on communication patterns and visible initiatives that firms adopt to seek endorsement and legitimacy in the eyes of the public or external actors (Hawn & Ioannou, 2016). The most notable external action is a firm’s ESG reporting. Although ESG reporting and ESG performance are intertwined, a firm may emphasize one strategy over the other for signaling purposes (García-Sánchez et al., 2021; Marquis & Qian, 2014). Recently, Luo et al. (2017) showed that firms adopt extensive reporting of overall ESG-related issues to accommodate competing institutional demands. We measure a focal firm’s ESG reporting and ESG performance using data from the same rating agency, precisely using *S&P Global ESG Scores.* The *S&P ESG Reporting Score* assesses firms’ sustainability reporting on a comprehensive set of 23 criteria distributed along the three ESG pillars. In the reporting score, S&P award points to a focal firm for any of the ESG criteria without normative assessment, simply based on the company’s ability to supply qualitative or quantitative information on a specific ESG issue (S&P, 2022). In contrast, the *S&P ESG Performance Score* measures how a focal firm is managing these 23 ESG criteria by assigning points to each criterion based on quantitative metrics that assess the substance of actions taken to manage these criteria. Both scores range from 0 to 100 points and are the weighted average of the three pillar scores constructed via the S&P Global Corporate Sustainability Assessment (CSA) methodology, an annual assessment of firms’ sustainability reporting and performance through survey and data mining (informed by the 1,000+ underlying data points gathered for each company; S&P, 2022). Because the S&P’s CSA methodology assesses the same set of ESG criteria for degree of reporting and the corresponding level of performance,^6^ it allows us to capture the degree of misalignment between advocated policies and implemented practices (Graafland & Smid, 2019). Supplemental Table A1 provides an example. A natural time lag exists between reporting and performance, therefore ESG decoupling is computed by subtracting the ESG performance score measured 1 year ahead (*t* + 1) from the ESG reporting score (*t*) (Bams & van der Kroft, 2025). Thus, positive (negative) scores on ESG Decoupling indicate higher (lower) levels of misalignment between ESG reporting and ESG performance score. To facilitate comparison with prior studies, we use two versions of the decoupling measure: one based solely on Environmental & Social (E&S) scores (*E&S Decoupling*, hereafter), and another that includes the governance pillar (*ESG Decoupling*, hereafter).

In order to provide external validity to our decoupling measure we performed a qualitative analysis for five firms in our sample (Amazon, Nike, PepsiCo, Starbucks, and Walmart) by comparing the results of the two ESG decoupling measures with information and performance measures about ESG issues that was reported by these five case companies in their sustainability report and other public sources of information. This qualitative analysis further corroborated the level of decoupling reflected through the two measures we used in the reported analyses. Due to space constraints, detailed information and supplementary data are included in the Supplemental Appendix.

### Independent Variable

To measure heterogeneity in shareholder proposal activism, we followed Harrison and Klein’s (2007) guidelines for conceptualization and measurement of within-unit diversity. Because we aim to measure how shareholder proposal activism “*differ from one another qualitatively that is, on a categorical attribute V*” (Harrison & Klein, 2007, p. 1204), we adopt a measure of diversity as variety^7^ combining two attributes; the identity of the shareholder initiating the proposal and the type of demand expressed in the proposal. The first attribute, which we refer to as shareholder activist identity, distinguishes “*the types of shareholder activists, as well as differences within each type*” (Chuah et al., 2024, p. 8) in terms of degree of adherence to social goals (i.e., SRI and non-SR; Gibson et al., 2022; Sikavica et al., 2020; Yan et al., 2019). Our second attribute captures the “*desired effect, type of impact, or expected outcome that the proposal seeks to achieve*” (Michelon et al., 2020, p. 5; i.e., SOSA and non-SOSA).

Consistent with previous studies (Baloria et al., 2019; Flammer et al., 2021; Goranova & Ryan, 2014; Sikavica et al., 2020), we coded the proposals as initiated by nine types of shareholder activists and then we further categorized them into two groups: SRI and non-SRI, to capture their relative adherence to social goals, as presented in Table 2, Panel B. Although the SRI and non-SRI classification for some types of shareholders is widely supported in previous literature (see Supplemental Table A2), the shareholder type “individual shareholders”^8^ have not been classified in this manner. We therefore retrieved the names of all “individual shareholders” who sponsored more than five proposals during the analysis period. These 32 “individual shareholders” (i.e., private persons) sponsored 89% of all the proposals initiated by this category in our sample. Using Google searches and LinkedIn, we identified “*what they do and how they describe who they are*” (Sikavica et al., 2020, p. 1238) to classify them as SRI or non-SRI shareholders. Table 2 shows that the majority of proposals in our sample are sponsored by non-SRI activists (59.99%), where the two largest categories are individual shareholders (25.51%) and public pension funds (13.24%). Of the proposals sponsored by SRI activists, 13.75% were initiated by SRI funds, 8.86% were initiated by faith-based funds, and 6.71% were initiated by special interest groups. This distribution is comparable to previous studies (Acharya et al., 2021).

Our second attribute relies on ISS’s description of each shareholder-initiated proposal, which allows us to identify the expected outcome put forward by the shareholder activist, and thereby the demand’s underlying logic (i.e., profit-oriented vs. social performance). Our coding approach followed guidelines suggested by Stemler (2001), which aims to apply explicit and replicable rules of coding to categorize words into fewer content categories. Building on the proposals’ description and previous research about different types of shareholder-initiated proposals (Acharya et al., 2021; Baloria et al., 2019; Michelon et al., 2020; Renneboog & Szilagyi, 2011), we created a list of keywords to differentiate proposals in terms of their expected outcome. More details are presented in Supplemental Table A3. These keywords were used to classify electronically each proposal into ten categories representing different environmental and social issues to which it refers (i.e., SOSA) and ten governance- and financial-related categories (i.e., non-SOSA). Two coding pretests were conducted manually by two authors on a random sample of shareholder-initiated proposals. After discussion and a few adjustments in the list of keywords, further pre-test coding was conducted, and the interrater reliability was .86. A total of 6,124 (95%) proposals were coded electronically using this list of keywords. Two of the co-authors independently manually coded the 322 shareholder-initiated proposals (5%) that had descriptions that did not include the keywords used for the electronic coding. Table 2, Panel C presents the distribution of shareholder-initiated proposals in terms of SOSA and non-SOSA. The proportion of non-SOSA (51.58%) is greater than SOSA (48.42%) proposals and aligns with previous literature (Acharya et al., 2021; Michelon et al., 2020). As expected, proposals concerning board of directors (BOD) and executive compensation (EXC) account for over 30% of non-SOSA proposals, while 40% of SOSA proposals address issues related to community (C) (15.20%), environmental (GHG, WM, W, and EM; 12.16%), disclosure of sustainability reports (SD; 6.10%), and product safety (P; 5.65%).

Table 2, Panel D presents a two-way tabulation, where the columns identify the number of SOSA and non-SOSA proposals (from Panel C), while the rows identify the relative adherence to social goals of shareholder activists sponsoring these proposals, SRI and non-SRI (from Panel B). Panel D shows that although non-SRI activists sponsor most of the non-SOSA proposals ( $\frac{2,334}{2,833}=$ 82.38%), they also sponsor many SOSA proposals ( $\frac{1,099}{2,890}=$ 38.03%). Similarly, whereas SRI activists sponsor most of the SOSA proposals ( $\frac{1,791}{2,890}=$ 61.97%), they also sponsor some non-SOSA proposals ( $\frac{499}{2,833}=$ 17.62%). This cross-tabulation illustrates the heterogeneity of shareholder proposal activism and how it can create shareholder pressures that are fragmented into different rationalized logics (Bromley & Powel, 2012; R. E. Meyer & Höllerer, 2016) via multiple and potentially competing demands enhanced by shareholder activists with different adherence to social goals (Acharya et al., 2021; DesJardine et al., 2023). Combining the two attributes we obtain the following four groups of shareholder-initiated proposals: (a) 2,334 non-SOSA by non-SRI, (b) 499 non-SOSA by SRI, (c) 1,099 SOSA by non-SRI, and (d) 1,791 SOSA by SRI. Based on these four groups of proposals, we adopt a measure of diversity as variety (Harrison & Klein, 2007) to capture the degree of proposal activism heterogeneity a targeted firm is exposed to, using a version of the Hirschman–Herfindahl Index, calculated with the following formula:

$$\mathrm{Identity}-\mathrm{Proposal}\;\mathrm{Het}._{i,t}\;=1-\sum \;p_{i,t}^{2}$$

where $p_{i}$ is the proportion of shareholder-initiated proposals in each of the four groups described above (see Table 2, Panel D) that firm *i* received at time *t.*^9^

We also computed three variations of the above index. The first reflects different logics arising from different activists’ identities (SRI vs. non-SRI) sponsoring the set of socially oriented proposals (SOSA) the focal firm received in a specific year, which we refer to *Identity Het. if SOSA*. The second contrasts different logics that arise from the type of demands expressed in proposals (SOSA vs. non-SOSA) but which are sponsored by shareholder activists that are not identified as socially responsible and presumably prioritize profit maximization, which we named *Proposal Het. by non-SRI*. Finally, we measure the degree of heterogeneity based only on the groups of proposals for which the activist’s identity conflicts with the nature of the demand, which we refer to *Diagonal Het.*, and represents the proposals shown in the upper right-hand and the lower left-hand quadrants of Table 2, Panel D. In Supplemental Table A4, we provide more details and numerical examples for these four indices of shareholder proposal heterogeneity.

### Control Variables

We selected a group of variables to control for factors that can potentially affect firms’ ESG policy-practice decoupling. First, because institutional investors tend to highly scrutinize firms’ disclosure and performance, we included *Institutional Ownership*, measured as the proportion of shareholdings held by all institutional investors in the firm’s total shareholders base (Flammer et al., 2021). To capture the extent to which firms’ governance mechanisms enhance discretion or impose limits on management (Baloria et al., 2019), we use the *Entrenchment* index developed by Bebchuk et al. (2009). Ranging from 0 to 6, this index is calculated as the sum of the following provisions: staggered boards, limits to shareholder bylaw amendments, poison pills, golden parachutes, and supermajority requirements for mergers and charter amendments. Based on the argument that a firm’s financial situation influences its capacity to engage in sustainable practices (García-Sánchez et al., 2021; Sauerwald & Su, 2019; Tashman et al., 2019), we also control for several financial attributes. *Size* is the natural logarithm of the firm’s total assets. *ROA* is earnings before interest, taxes, depreciation, and amortization divided by total assets. *Investment* is capital expenditures divided by total assets. *Organizational Slack* is cash and short-term investments divided by total assets. Finally, we controlled for the ratio of withdrawn proposals to the total number of proposals a firm has received in each year (*Withdrawn/Total*) and for the ratio of proposals omitted with approval by the SEC to the total number of proposals a firm has received in the same year (*Omitted/Total*). These two variables help control for, respectively, the alignment or friendliness of shareholder-initiated proposals to management and for the other extreme, when proposals are very hostile to the management (Clark et al., 2017). All control variables are winsorized at the 1st and 99th percentiles and measured 1 year preceding the measurement of our dependent variable.

## Results

### Descriptive Statistics

Table 3, Panel A presents descriptive statistics and correlation coefficients among the variables for the full sample, while Panel B presents descriptive and correlation coefficients for the subsample of targeted firms. In terms of heterogeneity in shareholder proposals, the minimum values are all zero, indicating that some targeted firms receive proposals that are similar both in terms of identity of shareholders activists and the nature of demands expressed in such proposals. The mean values are between 0.04 and 0.12, while the maximum values are either 0.5 or 0.75.

**Table 3.** Summary Statistics and Correlations.

Panel A: Full sample (*N* = 5,577)	1	2	3	4	5	6	7	8
(1) *E&S Dec*	1.00							
(2) *ESG Dec*	0.91	1.00						
(3) *Inst. ownership* (lag)	−0.09	0.00	1.00					
(4) *Entrenchment* (lag)	−0.19	−0.17	0.15	1.00				
(5) *Size* (lag)	0.16	−0.02	−0.26	−0.11	1.00			
(6) *ROA* (lag)	−0.06	−0.07	−0.00	−0.02	−0.04	1.00		
(7) *Investment* (lag)	0.02	−0.01	−0.09	0.01	0.11	0.13	1.00	
(8) *Org. Slack* (lag)	−0.04	−0.01	0.03	0.01	−0.25	0.08	−0.21	1.00
Min.	−66.05	−43.00	0.00	0.00	4.48	−0.32	0.00	0.00
Mean	1.80	12.11	0.83	3.55	8.73	0.14	0.04	0.14
Max.	73.56	66.04	1.00	6.00	11.98	0.40	0.25	0.74
*SD*	16.76	12.60	0.13	0.87	1.50	0.08	0.04	0.14

Panel B: Targeted firms (*N* = 3,457)	1	2	3	4	5	6	7	8	9	10	11	12	13	14
(1) *ESG Dec*	1.00													
(2) *E&S Dec*	0.92	1.00												
(3) *Identity-Proposal Het*. (lag)	0.12	0.06	1.00											
(4) *Identity Het*. if SOSA (lag)	0.12	0.08	0.64	1.00										
(5) *Proposal Het*. by non-SRI (lag)	0.12	0.08	0.68	0.55	1.00									
(6) *Diagonal Het*. (lag)	0.10	0.06	0.83	0.65	0.74	1.00								
(7) *Inst. Ownership* (lag)	−0.12	−0.03	−0.27	−0.23	−0.21	−0.20	1.00							
(8) *Entrenchment* (lag)	−0.24	−0.21	−0.12	−0.11	−0.11	−0.08	0.19	1.00						
(9) *Size* (lag)	0.20	0.04	0.46	0.34	0.35	0.36	−0.34	−0.20	1.00					
(10) *ROA* (lag)	−0.07	−0.06	−0.04	−0.02	−0.06	−0.06	0.03	−0.02	−0.22	1.00				
(11) *Investment* (lag)	0.03	0.00	0.11	0.11	0.06	0.11	−0.09	0.03	0.07	0.09	1.00			
(12) *Org. Slack* (lag)	−0.04	−0.01	−0.03	−0.03	−0.05	−0.03	0.06	0.00	−0.21	0.20	−0.24	1.00		
(13) *Withdrawn/Total* (lag)	0.01	0.02	−0.01	0.00	−0.02	0.01	0.04	0.03	−0.03	0.03	0.07	−0.03	1.00	
(14) *Omitted/Total* (lag)	0.04	0.02	0.06	0.02	0.04	0.05	0.00	−0.02	0.06	0.00	−0.01	0.02	−0.14	1.00
Min.	−66.05	−43.00	0.00	0.00	0.00	0.00	0.00	0.00	5.04	−0.32	0.00	0.00	0.00	0.00
Mean	2.06	11.21	0.12	0.04	0.05	0.07	0.81	3.56	9.39	0.14	0.05	0.13	0.12	0.10
Max.	73.56	63.75	0.75	0.50	0.50	0.50	1.00	6.00	11.98	0.40	0.25	0.74	1.00	1.00
*SD*	18.23	13.71	0.23	0.13	0.14	0.17	0.13	0.87	1.30	0.08	0.04	0.13	0.31	0.27

*Note. E&S Decoupling* is the difference between firms’ environmental and social reporting scores and respective performance scores. *ESG Decoupling* includes the reporting and performance scores for the governance pillar. *Identity-Proposal Het.* is the Herfindahl heterogeneity index combining both activist identities and proposal types of demands contained in the Panel D of Table 2. *Identity Het. if SOSA* is the Herfindahl heterogeneity index based on activist identities that sponsored the subset of SOSA proposals. *Proposal Het. by non-SRI* is the Herfindahl heterogeneity index based on the proposal types of demands for the subset of proposals sponsored by non-SRI activists. *Diagonal Het.* is the Herfindahl heterogeneity index based on non-SOSA proposals sponsored by SRI investors and SOSA proposals sponsored by non-SRI investors. *Institutional ownership* is the percentage of ownership by the largest shareholder classified as institutional. *Entrenchment* is Bebchuk et al.’s (2009) Entrenchment index. *Size* is the natural logarithm of market capitalization. *ROA* is earnings before interest, taxes, depreciation, and amortization over total assets. *Investment* is capital expenditures over total assets. *Org. Slack* is cash and equivalents over total assets. *Withdrawn/Total* is the ratio of resolutions that are withdrawn before voting over the the total number of resolutions a firm received in a year. *Omitted/Total* is the ratio of resolutions that are omitted by management over the total number of resolutions a firm received in a year. All continuous independent variables are winsorized at 1% and 99%. E&S = Environmental & Social; ESA = environmental, social, and governance; ISS = *Institutional Shareholder Services*; SOSA = socially oriented shareholder activism; SRI = socially responsible investing.

As expected, all shareholder proposals related variables (Panel B, targeted sample) are positively correlated with both *E&S Decoupling* and *ESG Decoupling*. The correlation coefficients for the control variables indicate that targeted firms that are larger, less profitable, and less liquid tend to show higher *E&S* and *ESG Decoupling*. As expected, *Institutional ownership* and *Entrenchment* are negatively correlated with *E&G Decoupling* and *ESG Decoupling*. Finally, the variables measuring the proportion of *Withdrawn* and SEC *Omitted* proposals are both positively correlated with *E&S* or *ESG Decoupling*. Only 11% of all shareholder proposals are withdrawn, and only 10% are omitted by management.

### Empirical Model

To examine the associations between, heterogeneity in shareholder proposal activism and decoupling, we used ordinary least squares (OLS) to estimate the model represented by Equation 1. Following standard practice in the literature, standard errors are clustered at the firm level.

(1) $$\begin{array}{c} ESG\;Decoupling_{i,t}=\;\beta_{1}+\beta_{2}\times Sh\_Activism\;Het_{i,t-1}\\ +\;\beta_{3}\times Institutional\;Own_{i,t-1}\\ +\beta_{4}\times Entrenchment_{i,t-1}+\;\beta_{5}\times Size_{i,t-1}\\ +\beta_{6}\times ROA_{i,t-1}+\beta_{7}\times Investment_{i,t-1}\\ +\;\beta_{8}\times Org.\;Slack_{i,t-1}+\beta_{9}\times Withdraw/Total_{i,t-1}\\ +\beta_{10}\times Omitted/Total_{i,t-1}\;\\ +Year\;FE\times Industry\;FE+\epsilon_{i,t} \end{array}$$

where *Sh_Activism Het* is either *Identity-Proposal Het, Identity Het if SOSA, Proposal Het by non-SRI*, or *Diagonal Het. Year FE* and *Industry FE* indicate fixed effects of year and industry, respectively. To control for potential unobservable yearly shocks in the dependent variables at the industry level, we also included the interactions between year and industry dummies as controls. To mitigate simultaneity concerns, all independent variables are lagged by 1 year.

Our first hypothesis predicts that firms exposed to more heterogeneous shareholder proposal activism will present greater ESG decoupling due to competing shareholder demands enacted from financial and social logics. In Table 4, we present the results for *E&S Decoupling* and *ESG Decoupling* as dependent variables, using our four measures for shareholder proposal activism heterogeneity as independent variables. As expected, we observe a strong and positive association between all four variables and *E&S* and *ESG Decoupling.* The coefficients in columns 1 to 8 are all positive, ranging from 4.81 to 8.26 (*t*-stats range 2.53–3.45, *p* < .05). We used columns 1 and 5 to estimate the marginal mean of *E&S* and *ESG Decoupling*. Increasing *Identity-Proposal Het.* from 0 to 0.75 in column 1 leads to an increase in the predicted mean of *E&S Decoupling* from 1.37 to 5.60, representing an almost threefold increase (4.23 points). When *ESG Decoupling* is the dependent variable (column 5), the rise is less substantial but remains important, with predicted mean increasing from 10.62 to 14.23, representing an increase of 34%.

**Table 4.** Regression Results for ESG Decoupling on Proposal Heterogeneity, Standard Errors Clustered by Firm, Targeted Sample.

Independent variables	Dependent variable: E&S Decoupling				Dependent variable: ESG Decoupling			
	1	2	3	4	5	6	7	8
*Identity-Proposal Het*. (lag)	5.63*** [2.86]				4.81*** [2.87]			
*Identity Het*. if SOSA (lag)		8.26** [2.53]				8.23*** [3.11]		
*Proposal Het*. by non-SRI (lag)			8.25*** [2.85]				8.10*** [3.33]	
*Diagonal Het*. (lag)				7.11*** [3.22]				6.46*** [3.45]
*Inst. Ownership* (lag)	−9.66** [−2.39]	−9.68** [−2.39]	−9.79** [−2.44]	−10.04** [−2.48]	−5.25 [−1.62]	−5.12 [−1.60]	−5.24* [−1.65]	−5.54* [−1.71]
*Entrenchment* (lag)	−0.47 [−0.68]	−0.48 [−0.70]	−0.47 [−0.68]	−0.47 [−0.68]	−0.11 [−0.20]	−0.12 [−0.22]	−0.11 [−0.20]	−0.11 [−0.20]
*Size* (lag)	2.31*** [5.17]	2.50*** [5.72]	2.47*** [5.52]	2.45*** [5.56]	0.08 [0.22]	0.21 [0.58]	0.19 [0.50]	0.18 [0.50]
*ROA* (lag)	7.28 [1.15]	7.04 [1.12]	7.66 [1.21]	7.64 [1.21]	2.32 [0.45]	2.02 [0.39]	2.64 [0.51]	2.62 [0.51]
*Investment* (lag)	12.95 [0.80]	13.56 [0.83]	14.39 [0.90]	13.60 [0.84]	6.63 [0.52]	6.98 [0.55]	7.82 [0.63]	7.12 [0.56]
*Org. Slack* (lag)	5.88 [1.40]	6.35 [1.51]	6.40 [1.51]	6.05 [1.44]	3.38 [1.04]	3.73 [1.16]	3.78 [1.16]	3.49 [1.07]
*Withdrawn/Total* (lag)	1.77* [1.84]	1.77* [1.85]	1.81* [1.89]	1.74* [1.80]	1.44* [1.95]	1.43* [1.96]	1.47** [2.01]	1.40* [1.91]
*Omitted/Total* (lag)	1.53 [1.26]	1.68 [1.38]	1.60 [1.31]	1.55 [1.27]	0.36 [0.40]	0.49 [0.54]	0.41 [0.45]	0.38 [0.41]
Constant	−24.03*** [−3.35]	−25.68*** [−3.60]	−25.27*** [−3.53]	−24.94*** [−3.48]	6.67 [1.12]	5.41 [0.91]	5.80 [0.97]	5.99 [1.01]
*R*^2^-adjusted	0.263	0.262	0.263	0.263	0.213	0.214	0.215	0.214
Observations	3,457	3,457	3,457	3,457	3,457	3,457	3,457	3,457

*Note*. See variables definition at the bottom of Table 3. Standard errors clustered by firm. Year fixed effects and year-industry fixed effects included. E&S = Environmental & Social; ESG = environmental, social, and governance; SOSA = socially oriented shareholder activism.

* *p* < .1. **p < .05. ***p < .01.

In columns 2 to 4 and 6 to 8, we included the more stringent variables of shareholder proposals heterogeneity, which captures the heterogeneity in terms of identity of shareholder activists (i.e., SRI or non-SRI) sponsoring the SOSA proposals (*Identity Het. if SOSA*), the heterogeneity in the type of proposals (SOSA or non-SOSA) sponsored by shareholder activists that are not identified as socially responsible investors (non-SRI) *(Proposal Het. by non-SRI)*, and the heterogeneity index computed based on the two subgroups of proposals that comprise conflicting logics enacted from proposal’s sponsor’s identity and type of demand (*Diagonal Het*.). All coefficients are both positive and significant, resulting in significant increases in the predicted means of the dependent variables. For instance, increasing *Diagonal Het.* from 0 to 0.5 leads to an increase in the predicted mean of *E&S Decoupling* of around 2.30 times, while *ESG Decoupling* increases around 30%. Taken together, these results support our argument that the greater the heterogeneity reflected in the identities of shareholders activists (SRI and non-SRI) and the type of demands (SOSA and non-SOSA) expressed in these proposals, the greater the need for targeted firms to respond with a misalignment between advocated policies and implemented practices.

To test Hypothesis 2, which predicts that decoupling is more persistent within firms exposed to more heterogeneous shareholder proposal activism, we estimate a partial adjustment model (i.e., Speed of Adjustment [SOA]). This approach has been widely used to examine firms’ persistent behavior (Flannery & Hankins, 2013; Öztekin & Flannery, 2012). We evaluate the persistence of decoupling by comparing the pace with which firms revert to the expected level of *E&S* and *ESG Decoupling.* Because our prediction focuses on the degree of heterogeneity of shareholder proposal activism, and not on the simple presence of shareholder proposal activism (i.e., being a target), we compare the persistence of decoupling between targeted firms with and without heterogeneous shareholder-initiated proposals.

Following Flannery and Hankins (2013), we start by defining a partial adjustment model and determining the firm’s expected level of decoupling (i.e., $ESG\;Decoupling_{i,t}^{*}$ ). We follow previous literature and assume that firms deviate from their expected outcome level, which in turn can be explained by observed and unobserved firm-level factors. Thus, to estimate $ESG\;Decoupling_{i,t}^{*}$ , we regress *E&S* and *ESG Decoupling* on the same set of control variables included in Equation 1 to extract the fitted values of *E&S* and *ESG Decoupling*, which are used as a proxy for the unobserved expected level of decoupling. Accordingly, the partial adjustment model is represented by Equation 2. To obtain the SOA ( λ ), we calculate one minus the estimated coefficient of $ESG\;Decoupling_{i,t-1}$ . Given that OLS and fixed-effect models yield biased estimates of λ (Flannery & Hankins, 2013), Equation 2 is estimated using the system generalized method of moments (GMM-Sys).^10^

(2) $$\begin{array}{c} ESG\;decoupling_{i,t}=\left(1-\;\lambda \right)\times \;ESG\;decoupling_{i,t-1}\\ +\lambda \times \left(\gamma X_{i,t}+Industry\;FE+Year\;FE\right)+\;\delta_{i,t} \end{array}$$

where *Year FE* and *Industry FE* indicate fixed effects of year and industry, respectively. *X* is a vector containing the control variables included in Equation 1. The results are presented in Table 5, using sub-samples based on our four measures of heterogeneity in shareholder proposal activism. In Panel A, the dependent variable is *E&S Decoupling*, while Panel B presents the results for *ESG Decoupling*.

**Table 5.** ESG and E&S Decoupling Persistence Tests with Robust Standard Errors, Estimated by Sys-GMM.

Test outputs	*Identity-Proposal Het.*		*Identity Het. if SOSA*		*Proposal Het. by non-SRI*		*Diagonal Het.*	
	(1)	(2)	(1)	(2)	(1)	(2)	(1)	(2)
	Het. = 0	Het. > 0	Het. = 0	Het. > 0	Het. = 0	Het. > 0	Het. = 0	Het. > 0
Panel A: E&S Decoupling								
E&S Decoupling (lag)	0.62*** [7.35]	0.79*** [13.74]	0.68*** [10.63]	0.67*** [5.63]	0.64*** [11.51]	0.74*** [13.02]	0.66*** [8.99]	0.75*** [12.15]
Observations	1,054	1,433	1,752	735	1,553	934	1,183	1,304
AR(1)	−5.67	−8.68	−5.53	−3.64	−7.24	−5.83	−6.31	−8.39
AR(1): *p*-value	.00	.00	.00	.00	.00	.00	.00	.00
AR(2)	1.13	−0.21	1.26	−0.51	1.30	−0.81	1.51	−0.63
AR(2): *p*-value	.26	.83	.21	.61	.19	.42	.13	.53
Hansen	22.80	18.05	27.70	26.73	55.07	47.96	22.34	17.31
Hansen *p*-value	.25	.52	.53	.59	.09	.24	.22	.50
Nr. instruments	60.00	59.00	71.00	66.00	84.00	80.00	60.00	56.00
Diff. (1) − (2) (*t*-stat)	−0.16 (−54.01)		0.01 (1.71)		−0.10 (−41.86)		−0.09 (−31.61)	
Panel B: ESG Decoupling								
ESG Decoupling (lag)	0.70*** [9.31]	0.78*** [14.55]	0.70*** [15.92]	0.69*** [11.86]	0.72*** [12.92]	0.77*** [11.42]	0.72*** [10.75]	0.77*** [14.91]
Nr. observations	1,054	1,433	1,752	735	1,553	934	1,183	1,304
AR(1)	−5.47	−9.06	−7.68	−5.84	−7.59	−7.20	−6.12	−8.78
AR(1): *p*-value	.00	.00	.00	.00	.00	.00	.00	.00
AR(2)	0.94	−0.08	1.12	−0.25	1.04	−0.45	1.29	−0.39
AR(2): *p*-value	.34	.94	.26	.80	.30	.65	.20	.69
Hansen	23.43	16.65	49.11	40.68	23.65	17.33	21.30	18.71
Hansen *p*-value	.17	.55	.07	.27	.17	.50	.26	.41
Nr. instruments	59.00	58.00	78.00	73.00	60.00	56.00	60.00	56.00
Diff. (1) − (2) (*t*-stat)	−0.09 (−33.03)		0.01 (2.70)		−0.05 (−19.50)		−0.05 (−21.29)	

*Note*. All control variables included but untabulated. See variables definition at the bottom of Table 3. Year and industry fixed effects are included. We treated the lagged dependent variable as endogenous and used all lagged values as instruments. We treated the control variables as predetermined and used the lags *t* −1 and *t* − 2 as instruments. We treated all year and industry fixed effects as exogenous. We use the orthogonal transformation for the predetermined variables and collapse the instruments to avoid proliferation.

* *p* < .1. **p < .05. ***p < .01.

In Panel A, the coefficient of the lagged value of *E&S Decoupling* when firms receive heterogeneous proposals (i.e., when *Identity-Proposal Het.* is greater than zero) is 0.79, while the coefficient of the lagged value of *E&S Decoupling* when firms receive homogeneous activism (i.e., when *Identity-Proposal Het.* is zero) is 0.62. That is, the decoupling SOA of firms targeted with homogenous shareholder proposals is significantly smaller, suggesting that E&S policy-practice decoupling persists for a longer time in firms targeted with heterogeneous proposals compared to firms targeted with homogeneous proposals. The *t*-statistics of the difference is larger than 3, implying that it is statistically different than zero.

We find similar results when we use two of the three alternative proxies of activism heterogeneity (i.e., *Proposal Het by non-SRI* and *Diagonal Het.*) with significant statistical differences between the persistence of *E&S Decoupling* of targeted firms exposed to homogeneous proposals compared with firms exposed to heterogeneous proposals. When we split the sample of firms receiving heterogeneous versus homogeneous proposals as measured by *Identity Het. if SOSA*, we find more similar coefficients, implying a small and unexpected positive difference, though much less substantial and statistically significant compared to the other three measures. Overall, the results in Table 5 suggest that the average persistence of *E&S Decoupling* in firms receiving heterogeneous shareholder-initiated proposals is roughly 0.09 points smaller, but the difference is as large as 0.16 points, with *p*-values ranging from .000 to .088. This implies that the average firm receiving heterogeneous proposals takes around 0.9 years more to return to the expected level of *E&S Decoupling* than those firms receiving homogeneous proposals. More precisely, the average coefficient is 0.75 when firms receive heterogeneous proposals and 0.68 when they receive homogeneous proposals. These coefficients imply that the SOA is 0.25 and 0.32, respectively. Hence, the results suggest that, on average, firms targeted by heterogeneous activism close 25% of the gap between the current and expected levels of decoupling per year (i.e., the gap is closed in approximately 4 years), whereas firms targeted by homogeneous activism close 32% of the gap annually (i.e., the gap is closed in about 3.1 years). Taken together, the evidence presented in Table 5, Panels A and B, supports our Hypothesis 2, whereby the heightened state of ambiguity caused by intra-institutional complexity reflected in heterogenous shareholder proposal activism, leads to greater persistence of decoupling.

### Supplementary Analysis

We test the robustness of our results in several ways. First, our main analyses focused on the subsample of firms that received shareholder-initiated proposals, raising concerns about selection bias. To overcome this issue, we estimate Equation 1 using the full sample (5,577 firm-year observations) but including interaction terms between a dummy variable (*Targeted*) and all independent variables. The results are presented in Supplemental Table A5, and show that the coefficients for each of the four interaction terms are positive and mainly significant, which corroborates the main analyses. *E&S* and *ESG Decoupling* is higher among firms with heterogenous shareholder proposal activism, relative to either targeted firms with homogenous proposals or non-targeted firms.

To address the concern that firms exposed to heterogeneous shareholder proposal activism may be fundamentally different from firms that are not, which in turn may affect their response in terms of decoupling, we created a balanced sample using an entropy balancing technique (DesJardine et al., 2023; Hainmueller, 2012). We reweight observations to balance the first two moments of the distribution of all covariates of a subsample containing targeted firms with heterogeneous activism to those of the subsample containing targeted firms with homogeneous activism. The results (Table A6, Panel A and Table A8 in the Supplemental Appendix) corroborate our main results in Table 4, as we find only positive and significant coefficients. Similarly, the underlying differences between firms targeted by heterogenous and homogenous shareholder proposal activism might also impact the results about the persistence of decoupling. To address this issue, we also estimate Equation 2 using the subsamples of firms balanced by the entropy balancing technique (see Supplemental Appendix Tables A7, A9, and A10). The results corroborate those in Table 5, as we find that the coefficients of *E&S* and *ESG* decoupling are larger in the sample of firms targeted by heterogeneous shareholder proposal activism than in the subsample of firms targeted by homogeneous activism.

In our main tests of Hypothesis 1 (Table 4), we use the lagged values of heterogeneity in shareholder proposal activism, which may raise concerns about possible year-specific clusterization of proposals affecting our results. In other words, firms receive many proposals in a given year followed by years without shareholder-initiated proposals. To account for this concern, we calculate a 3-year moving window average of our independent variables of interest and re-estimate Equation 1. The results (Supplemental Appendix Table A6, Panel B) corroborate those of Table 4. Less sustainable firms are more likely to decouple (García-Sánchez et al., 2021) and may attract more activism from shareholders. We therefore conduct three tests to ensure that our results are not influenced by reverse causality. More precisely, we estimate our model excluding sample firms that received more than five proposals each year,^11^ sample firms in the top highest 5% values of heterogeneous activism, and the bottom 5% of sample firms in terms of past ESG performance using 1-year lagged data. The coefficients are presented in Supplemental Appendix Table A6, Panels C, D, and E, respectively, and suggest that reverse causality is not driving our results.

Previous literature suggests that ESG risks and opportunities are industry-specific, which may influence the level of shareholder activism and the nature of proposals these firms are exposed to (Denes et al., 2017). Hence, we estimated our model using industry mean adjusted values of our independent variables and including a dummy variable identifying firms operating in social and environmental sensitive industries^12^ (Schreck & Raithel, 2018). Again, the results reported in the Supplemental Table A6, Panels F and G, confirm those of our primary analyses (Table 4). As explained above in our methods, we measured *E&S* and *ESG Decoupling* using firms’ performance scores 1 year ahead of reporting scores. However, it is plausible that ESG-related initiatives may take longer to generate a measurable impact on ESG performance. To address this potential issue, we recompute our decoupling variables using performance scores measured 2 years ahead of corresponding reporting scores and further test the robustness of our findings using a 3-year average performance score to calculate our decoupling measure and firm fixed effects. These alternative results, which are presented in the Supplemental Table A6, Panels H and I, are qualitatively similar to our main results presented in Table 4. Further, competing expectations from institutional shareholders may create more pressure on targeted firms than differing demands from less influential shareholder activists. We therefore recalculated our measures of activism heterogeneity, restricting to proposals sponsored by institutional investors and re-estimated our main model (Table 4). The results, summarized in Supplemental Appendix Table 6, Panel J, are consistent with the main findings, suggesting that whether heterogenous demands are enacted from institutional shareholder activists or other types of shareholder activists does not impact our results. Finally, we estimate our model using only proposals that were voted on (Supplemental Appendix Table A6, Panel K). Again, we corroborate our main results.

## Discussion

Shareholder activists with different identities and interests are leveraging their financial logic embedded in their ownership position and voting rights to support or oppose^13^ responsible environmental and social practices (Gond & Piani, 2013). Because environmental and social performance goals are perceived to belong to a distinct logic and are misaligned with the central premises of financial logic, we argue that shareholder proposal activism which simultaneously prioritizes financial and social performance goals, exposes firms to intra-institutional complexity (R. E. Meyer & Höllerer, 2016; Yan, 2020; Yan et al., 2019). An extensive body of research has examined the effects of shareholder activism by focusing either on a particular type of activism—socially versus non-socially oriented—or by treating differences in shareholder activist identities and demands as equivalent (Denes et al., 2017; Goranova & Ryan, 2014). We extend this prior research by considering how the heterogeneity in shareholder activists’ demands and identities may together affect the occurrence and persistence of firms’ symbolic or substantive responses (García-Sánchez et al., 2021; Graafland & Smid, 2019; Herman et al., 2024). We maintain that when shareholders simultaneously demand actions underpinned by both profit-oriented and social performance logics, the scrutiny becomes dispersed and makes it more difficult to differentiate between symbolic and substantive actions, leading to greater and more persistent decoupling.

Our empirical analysis supports our predictions. We show that shareholder proposal activism embraces multiple logics, reflected in both activists’ relative adherence to social goals and the type of demands. More precisely, although our sample shows a balance between SOSA and non-SOSA proposals, more than 40% of SOSA proposals are sponsored by traditional shareholder activists, or at least shareholders who do not publicly adhere to social performance goals (i.e., non-SRI). The various analyses provide strong evidence that when shareholder proposals blend profit-oriented and social performance goals to a greater extent, firms are more likely to respond with symbolic actions, for a consequential misalignment between advocated ESG policies and implemented practices.

Our results also demonstrate significant differences in the persistence of ESG policy-practice decoupling between targeted firms exposed to homogeneous as opposed to heterogeneous shareholder proposal activism. While ESG policy-practice decoupling is not a long-lasting strategy in firms targeted by homogenous shareholder proposals, for targeted firms that experience heterogenous shareholder proposal activism, ESG policy-practice decoupling is significantly more persistent. As R. E. Meyer and Höllerer (2016) emphasize, decoupling is probably a strategy for gaining time to find out how shareholder expectations may evolve or to ensure that future lines of action remain available. By examining simultaneously, the different orientations and competing demands of shareholder activists, we advance research on organizational responses to intra-institutional complexity (R. E. Meyer & Höllerer, 2016; Yan et al., 2019, 2021). Further, we answer calls to “*empirically address the heterogeneity of shareholder activism*” (Goranova & Ryan, 2014, p. 1256) and for researchers to focus on firms’ symbolic or substantive responses to institutional pressures (Graafland & Smid, 2019). Our findings, therefore, have several theoretical and practical implications.

### Theoretical Implications

Although previous research has shown that firms symbolically respond to conflicting demands from different stakeholders (i.e., customers, employees, community, regulators; Crilly et al., 2012; Testa et al., 2018), much less is known about how firms behave when incompatible prescriptions arise and compete within the same stakeholder group. Our conceptualization of heterogeneity in shareholder proposal activism as a source of intra-institutional complexity that not only prompts decoupling but also leads to its persistence directly addresses Greenwood et al.’s (2011, p. 350) calls to explore various sources of institutional complexity and to examine “*the motivations behind decoupling as a response to institutional complexity*.” In addition, the persistence of decoupling is an area that has been relatively unexplored in previous research (Boxenbaum & Jonsson, 2017; Herman et al., 2024), and one highlighted as an important topic for inquiry (Hawn & Ioannou, 2016). Relatedly, our findings support our arguments that demands rooted in varying degrees of emphasis in financial versus social goals (i.e., SOSA, non-SOSA) and channeled by the same constituency with different investment orientations (SRI, non-SRI), create acute ambiguity in defining and prioritizing competing logics. This ambiguity blurs the boundaries between these competing demands, making it more difficult for firms to resolve or deflect scrutiny. In doing so, our study offers a refinement to the theoretical distinction between intra- and inter-institutional complexity (R. E. Meyer & Höllerer, 2016). Thus, our findings resonate with the need to build a more complete theory of organizational responses to stakeholder demands (Haack & Schoeneborn, 2015; Pache & Santos, 2013).

Second, despite widespread academic interest in the influence of shareholder activism on firm-level outcomes (Chuah et al., 2024), our study shifts attention to the extent to which responses are more symbolic than substantial. Our results suggest that heightened ambiguity in shareholder expectations—rooted in the divergence between financial and social logics—increases the flexibility firms have concerning courses of action, which in turn can affect the cost-benefit balance of shareholder activism. As Bromley and Powell (2012) emphasize the increasing pressure for transparency and accountability has engendered more widespread organizational symbolism. As such, our results contribute to our understanding of why firms may place legitimacy at risk to respond to shareholder activism, and the conditions that motivate (or inhibit) such symbolic behavior over longer periods of time.

Third, our research extends key insights from prior studies on ESG decoupling (García-Sánchez et al., 2021; Graafland & Smid, 2019; Herman et al., 2024). Whereas previous research has shown that ESG decoupling is moderated by external monitoring (García-Sánchez et al., 2021) or the quality of ESG initiatives (Graafland & Smid, 2019), our findings emphasize the role of intra-institutional dynamics in fostering decoupling behavior. Additionally, we build on Gond and Piani’s (2013) work by highlighting how heterogeneous shareholder demands amplify the legitimacy challenges firms face in implementing responsible business practices and gaining external credibility, leading to symbolic management practices.

Finally, and more broadly, we also contribute to the ongoing debate on whether and how shareholders impact non-equity stakeholder interests. Our findings complement the work of DesJardine et al. (2023), suggesting that the impact of proposal activism on the interests of non-equity stakeholders is contingent upon the type of shareholders who file the socially oriented proposals. By decomposing shareholder proposal activism in terms of shareholder activists’ relative adherence to social performance goals (Sikavica et al., 2020) and the proposals’ expected outcomes (Michelon et al., 2020), we reveal two different and potentially combinatory sources of heterogeneity, supporting empirically the idea that shareholder activism comprises different “*interests, demands, and identities*” (Goranova & Ryan, 2014, p. 1260). Further, by examining proposals sponsored by all types of shareholder activists (i.e., not only institutional investors), we address concerns that the most research on shareholder activism has concentrated on certain types of activists (e.g., institutional investors and hedge funds), while other shareholder activists and their competing and simultaneous demands have been overlooked (Chuah et al., 2024). This is important as it provides new insights into how external governance mechanisms intended to enhance shareholders’ voice and legitimacy can have the unintended consequence of facilitating a firm’s symbolic management (Westphal & Zajac, 2001).

### Practical Implications

Our study contributes to the normative debate on whether further shareholder empowerment is an effective means to mitigate environmental and social issues. Our results provide guidance to activist investors about how their actions impact the ways firms address environmental and social demands. More specifically, it demonstrates how management use decoupling to balance financial/social trade-offs (Battilana et al., 2022) that emerge from competing, and sometimes conflicting shareholder expectations. This is a timely and contemporaneous topic, given the recent SEC amendment to the shareholder proposal rules (Rule 14a-8), designed to enhance the ability of shareholders to exercise their voting rights. Among other things, this policy reduced management’s ability to omit shareholder proposals from the voting ballot, which may create greater discretion and further room for symbolic responses when firms react to heterogeneous shareholder activism.

### Limitations and Future Research

Despite the richness of our dataset and context, our study focuses on shareholder proposal activism, one specific form of shareholder activism that signals formal and public disapproval of the misalignment between firm behavior and societal expectations. However, shareholder activism may encompass other activities beyond proposals, including behind-the-scenes engagement, proxy contests, or media campaigns (Chuah et al., 2024; Denes et al., 2017). Exploring the heterogeneity in other types of shareholder activism and their distinct influence on decoupling behavior would augment our findings and help provide further understanding of how shareholder activism shapes symbolic versus substantial ESG performance.

In addition, not all shareholder activists are the same in terms of their power to influence firm actions. Although we control for institutional shareholder ownership, our dataset does not permit a detailed examination of ownership levels for all activist types in our sample, and therefore, we are not able to explicitly account for the power dynamics among different types of shareholder activists. By addressing this limitation, future research could extend our findings by investigating how variations in salience or ownership levels among shareholders influence firms’ strategic responses to activism. We acknowledge that the ideological and regulatory context around ESG has shifted in recent years, including growing political backlash. These changes may influence how investors describe their engagement goals, particularly those who previously self-identified as responsible investors. While our coding was conducted in 2022, prior to the visible escalation of anti-ESG sentiment, and self-identification was used to code only individual shareholders, future research could examine how such ESG backlash has shaped investor identity and adherence to social issues across different investor types. In line with Clark et al. (2024), we also recognize that global social movements (i.e., #MeToo, Black Lives Matter, and Greta Thunberg’s Fridays for Future) shape how firms perceive and respond to SOSA. While our data covers a period when these movements gained traction (2013–2021), our design does not allow for isolating their effects. Future studies could explore this intersection more directly. Another interesting extension of our study would be to investigate how intra-institutional complexity within other stakeholder groups, such as employees, customers, or non-governmental organizations, could also shape firms’ strategic decisions and responses to external pressures.

Our analysis relies on a specific pair of ESG reporting and performance ratings, which may reflect underlying assumptions about ESG risks and how firms address them (Bams & van der Kroft, 2025). Importantly, ongoing debates persist over whether ESG ratings should emphasize corporate impact or exposure to financially material ESG risks (Eccles & Stroehle, 2020). Future research could extend our study by comparing how impact- versus risk-oriented ratings shape assessments of ESG performance and influence stakeholder perceptions. Additionally, our study is situated within the U.S. context, where the institutional, regulatory, and cultural environment uniquely shapes shareholder activism and firms’ responses to it. Countries with different governance structures, stakeholder-oriented systems, or regulatory regimes may experience distinct dynamics in how firms reconcile societal expectations with shareholder demands (Sauerwald et al., 2016). Future research could explore these phenomena in non-U.S. contexts to assess how institutional environments influence shareholder activism and decoupling behavior.

Lastly, our study covers a 9-year period (2013–2021). Longer time horizons could provide additional insights into shifts in the prevalent logics underpinning shareholder activism and their effects on firms’ symbolic and substantive responses over time (R. E. Meyer & Höllerer, 2016; Yan, 2020). Longitudinal studies could also capture evolving dynamics between activists and firms (Chuah et al., 2024), offering a more comprehensive understanding of the temporal aspects of ESG decoupling and corporate adaptation to shareholder pressures.

## Conclusion

In this study, we demonstrate that heterogeneity in shareholder proposal activism—reflected in the different identities of shareholder activists and the competing nature of their demands—intensifies the ambiguity and trade-offs faced by targeted firms, which, in turn, affects the occurrence and persistence of policy-practice decoupling. These findings deepen our understanding of how firms navigate the complexities of competing demands from a diverse set of shareholder activists. We hope this study will advance research on firms’ responses to institutional pressures and complexity in ways that can assist executives and investors in managing the tension between financial and social performance.

## Supplemental Material

sj-docx-1-bas-10.1177_00076503251357372 – Supplemental material for Competing Voices, Persistent Gaps: Shareholder Proposal Activism and ESG Decoupling Supplemental material, sj-docx-1-bas-10.1177_00076503251357372 for Competing Voices, Persistent Gaps: Shareholder Proposal Activism and ESG Decoupling by Eduardo Schiehll, Henrique Castro Martins and Krista B. Lewellyn in Business & Society

## Notes

1. Shareholder-initiated proposals apply coercive power via formal mechanisms associated with shareholder rights. These include minimum ownership and eligibility requirements to introduce a proposal, the ex-ante consent by the Security and Exchange Commission (SEC) for the proposal, the public disclosure of a firm’s proxy ballot, and shareholders’ voting rights in annual meetings (Vasi & King, 2012).

2. In contrast to less institutionalized forms of stakeholder activism (e.g., boycotts, petitions, staged walkouts), shareholder-initiated proposals apply coercive power via formal mechanisms of shareholder rights. These, include ownership and eligibility requirements to introduce a proposal, the ex-ante consent by the Security and Exchange Commission (SEC) for the proposal, the public disclosure of a firm’s proxy ballot, and shareholders’ voting rights in annual meetings (Bates & Hennessy, 2010; Vasi & King, 2012).

3. Investment orientation refers to shareholders’ relative interests in nonfinancial outcomes, such as social and environmental externalities, and can influence how different types of shareholders promote or hinder the interests of non-equity stakeholders (DesJardine et al., 2023).

4. The analysis period began in 2013 to ensure consistency in the methodology used by S&P Global for calculating its ESG Reporting Score and ESG Performance Score. For more details, see S&P (2022).

5. Because we are primarily interested in the heterogeneity of demands initiated by shareholders, we include all proposals, including those that were withdrawn or omitted by the SEC. A similar approach is taken by Flammer et al. (2021), who cover only climate-change related activism. Although omitted and withdrawn proposals never come to a vote, they still represent shareholders’ expectations and pressure on the firm’s management (Acharya et al., 2021). In the robustness analysis (Supplemental Table A6, Panel E), we present the results of restricting our analysis to only proposals that came to a vote.

6. As shown by Berg et al. (2022), the data sources used to populate ESG ratings include public, quasi-public, private data. Since the different sources do not use similar methods, working with ESG ratings from different rating agencies brings inherent challenges such as completeness of data items, standardization of metrics, and measurement consistency. By using ESG reporting and ESG performance scores provided by the same rating agency, S&P Global ESG Scores, we minimize these problems.

7. Harrison and Klein (2007) distinguish three types of group diversity: separation, disparity, and variety. Separation captures positional differences (e.g., values or opinions), disparity reflects differences in resource levels (e.g., power or status), and variety refers to categorical differences (e.g., background or expertise). The latter is the focus of our study.

8. Denotes private persons who possess ownership stakes in a company. These shareholder activists are distinct from financial institutions or corporations and can include private investors, employees, or other non-institutional entities.

9. To illustrate, based on our example presented in the introduction, from the 17 SOSA proposals appearing on the 2020 Amazon Proxy Ballot, 9 proposals were initiated by SRI activists, while 8 proposals were initiated by non-SRI activists. Among the three non-SOSA proposals submitted to AMZON in the same year, one proposal was sponsored by an SRI activist. In this case, the calculated $Identity-Proposal\;Het_{i,t}$ -*I* for AMZON for that year equals 0.625: ( $1\;-[\;{\left(\frac{9}{20}\right)}^{2}+\;{\left(\frac{8}{20}\right)}^{2}+{\left(\frac{1}{20}\right)}^{2}+\;{\left(\frac{2}{6}\right)}^{2}$ ]).

10. In untabulated tests, we also estimate Equation 2 using Fixed Effects, GMM, and the Least Squares Dummy Variable Correction, all leading to similar results. For a review of dynamic panel models, see Flannery and Hankins (2013).

11. We exclude firms receiving more than five proposals, as they represent the top 10% of the distribution (i.e., the most heavily targeted firms) and may introduce reverse causality bias.

12. Food & Beverages; Household & personal products; Pharmaceutical & biotechnology; Chemicals; Pulp & Paper; Construction; Machinery; Automobile; Metal & mining; Oil & gas; and Utilities.

13. For recent examples, see: Are Shareholders Turning Against ESG? By Harlan Tufford, Voce President, MSCI Research. https://www.msci.com/www/esg-now/are-shareholders-turning/03919756686
