# Supplementary material for: Competing Voices, Persistent Gaps: Shareholder Proposal Activism and ESG Decoupling
Source: Bus Soc. 2025 Aug 20;65(6):1643–81. doi: 10.1177/00076503251357372 (PMC13241000; doi:10.1177/00076503251357372)
Supplement: sj-docx-1-bas-10.1177_00076503251357372 – Supplemental material for Competing Voices, Persistent Gaps: Shareholder Proposal Activism and ESG Decoupling [file sj-docx-1-bas-10.1177_00076503251357372.docx]

**Table A1.** Measurement of ESG Decoupling; illustrative example

| Year | KeyField | Weight  (%) | KeyField | ESG Performance^£^ | Score | KeyField | ESG Transparency & Disclosure^¥^ | Score | Decoupling |
| --- | --- | --- | --- | --- | --- | --- | --- | --- | --- |
| Amazon (AMZN) | | |  |  |  |  |  |  |  |
| 2021 | 331429 | 24 | 331428 | Environmental | 33.3 | 333201 | Environmental Reporting | 100 |  |
|  | 331431 | 28 | 331430 | Social | 20. | 333203 | Social Reporting | 33.5 |  |
|  | 331435 | 48 | 331434 | Governance & Economic | 24.10 | 333205 | Corporate Governance | 74.2 |  |
|  |  |  | 331427 | ESG Performance | 25.3 |  | ESG Reporting | 69.0 | **43.7** |
| Motorola Solutions Inc. (MSI) | | | |  |  |  |  |  |  |
| 2021 | 331429 | 31 | 331428 | Environmental | 46.6 | 333201 | Environmental Reporting | 100 |  |
|  | 331431 | 25 | 331430 | Social | 32.0 | 333203 | Social Reporting | 50.0 |  |
|  | 331435 | 44 | 331434 | Governance & Economic | 51.1 | 333205 | Corporate Governance | 61.8 |  |
|  |  |  | 331427 | ESG Performance | 44.9 |  | ESG Reporting | 70.7 | **25.8** |
| General Motors Company (GM) | | | |  |  |  |  |  |  |
| 2021 | 331429 | 31 | 331428 | Environmental | 81.6 | 333201 | Environmental Reporting | 93.0 |  |
|  | 331431 | 32 | 331430 | Social | 79.3 | 333203 | Social Reporting | 57.0 |  |
|  | 331435 | 37 | 331434 | Governance & Economic | 75.0 | 333205 | Corporate Governance | 71.12 |  |
|  |  |  | 331427 | ESG Performance | 78.4 |  | ESG Reporting | 73.5 | **-4.9** |

**^£^ESG Performance Score**: scores awarded between 0–100 points for quantitative metrics assessed on the quality and substance of values compared to a potential maximum-scoring response. For example, if a question asks for the precise number of female directors on a board (e.g., Question 1.1.4 Board Gender Diversity in this questionnaire), it may be scored relative to the total number of directors (e.g., Question 1.1.1 Board Structure in the same questionnaire). (S&P Global ESG Scores – Methodology, 2021)

**^¥^ESG Transparency & Disclosure Score**: scores awarded between 0–100 points for qualitative or quantitative information supplied without normative assessment of values. For example, if a question asks for the share of female employees in the workforce (e.g., Question 3.2.2 Workforce Breakdown: Gender in this questionnaire), points may be awarded for any given response reflecting a policy is in place or due to the company’s ability to supply this metric, indicating that this is something the company is actively tracking and reporting. (S&P Global ESG Scores – Methodology, 2021)

**A Case-base Analysis to validate our measures of ESG Decoupling**

In this appendix we analyse five firms in our sample, to validate our decoupling measure. We compare the level and trend of our decoupling measure with qualitative, granular disclosure and performance information reported by these five firms. This qualitative analysis supports our measure which, aims to capture the gap between policy statements that are not implemented or at least without substantial effect on relevant ESG metrics.

AMAZON

The mean value of ESG decoupling for Amazon is -3.93, indicating a negative and low level of decoupling in the period of analysis. ESG decoupling starts to increase in 2018, with maximum value of 29.36 in 2019. Looking more qualitatively at the information available from Amazon, we observe that it coincides with the firm’s first public commitment to a net-zero carbon pledge in 2018 (Amazon, 2019), while subsequent related performance metrics (GHG total emission and intensity) did not show improvement before 2021 (Amazon, 2023: 11).

<https://sustainability.aboutamazon.com/2019-sustainability-report.pdf>

<https://sustainability.aboutamazon.com/2023-amazon-sustainability-report.pdf>

NIKE

In the case of Nike, the mean value of decoupling is positive and shows an increasing trend over the period of analysis. Mean value is 10.53. The maximum levels of decoupling are observed in 2021 (15.76). Looking closer at Nike’s public disclosures, commitments to worker safety contrasted with the reality of unsafe conditions in some of its factories, as documented by events in Bangladesh (2013), Cambodia (2014), and Vietnam (2017) (FT, 2023), as well as metrics reported in Nike Impact reports (2023).

https://about.nike.com/en/impact?filter=DEI

Financial Times (2023). Asia factory workers press Nike over missing pay. <https://www.ft.com/content/b2fb7264-aea0-4941-845b-40289b0f0238>

PEPSICO

PepsiCo shows a mean value of ESG decoupling of 15.27. The trend goes from negative decoupling (2013 to 2015) to positive decoupling (2016 to 2021). Maximum level of ESG decoupling is observed in 2017 (45.36). The increasing level of decoupling apparently coincides with PepsiCo facing increased criticism for marketing sugary beverages to children and teenagers, contradicting its public commitments to healthier products (PepsiCo, 2017: 12-13). In its 2020 Sustainability Report, PepsiCo announced revised commitments to reduce sugar, fat, and salt in its products (PepsiCo, 2020: Focus Area Product, pages 1 and 2).

<https://www.pepsico.com/docs/default-source/sustainability-and-esg-topics/2017-sustainability-report.pdf?sfvrsn=60529676_2>

<https://www.pepsico.com/docs/default-source/sustainability-and-esg-topics/2020-sustainability-report.pdf?sfvrsn=bf192307_6>

STARBUCKS

The mean value of decoupling for Starbucks is 11.83 and shows an increasing trend over the period of analysis. The level of decoupling is relatively low before 2015 but increases remarkably afterwards, reaching 16.49 in 2016. Public disclosure shows that concerns about labor rights emerge in 2015, showing an ongoing gap between Starbucks' public commitments and workers’ actual experiences (Starbucks, 2015:8; 2019:8-10). Precisely, in 2015 and 2016, employee protests over low wages and poor working conditions in the U.S. led to unionization efforts at multiple locations (FT, 2021). In 2018, the company faced environmental criticism for its use of disposable cups (FT, 2019).

<https://www.ft.com/content/b3267082-04c0-4b39-98b8-ac8263848be9>

<https://www.ft.com/content/fe536926-ec73-11e9-a240-3b065ef5fc55>

2019 Global Social Impact Report (<https://content-prod-live.cert.starbucks.com/binary/v2/asset/143-71920.pdf>)

2015 Global Social Impact Report (<https://content-prod-live.cert.starbucks.com/binary/v2/asset/143-71939.pdf>)

WALMART

In the case of Walmart, ESG decoupling is on average high and positive throughout the period, with a mean value of 20.89. The lowest level of ESG decoupling is observed in 2015, 8.15, the year in which the company revised its reduction targets to align with SBTi (18% by 2025 relative to 2015 levels) (SBTi, 2016). Publicly available information indicates Walmart faced difficulties achieving their ambitious emission goal (Walmart, 2021), which is reflected in our decoupling measure, and which shows an increasing trend after 2015.

<https://sciencebasedtargets.org/blog/walmarts-science-based-target-a-game-changer>

<https://corporate.walmart.com/content/dam/corporate/documents/esgreport/cdp-response-archive/cdp-climatechange2021.pdf>

**Table A2.** Categories and Related Literature supporting our classification of Shareholder Activists

| **Category^[[1]](#footnote-1)^** | **Related literature supporting classification** | **Examples (name of Shareholder Activists)** |
| --- | --- | --- |
| **SRI Activists** | | |
| Faith-based funds | Acharya, Gras & Krause (2021); Hadani, Doh & Scheider (2018); Goranova & Ryan (2014:1233); Sikavica, Perrault & Rehbein (2020) | Benedictine Sisters of Chicago; Catholic Health Initiatives; Mercy Investment Services; United Methodist. |
| SRI funds | Acharya, Gras & Krause (2021), Goranova & Ryan (2014), Desjardine, Zhang & Shi (2023), Yan *et al*. (2019) | Boston Common Asset Management; Green Century Capital Management; Pax World Mutual Funds; Trillium Asset Management. |
| Special interest groups | Benton (2017); Hadani, Doh & Scheider (2018); Guercio & Woidtke (2019); Sikavica, Perrault & Rehbein (2020); DesJardine, Zhang & Shi (2023) | As You Sow Foundation; Oxfam America; People for the Ethical Treatment of Animals. |
| Union pension funds | Rehbein, Wadock & Gaves (2004); Acharya, Gras & Krause (2021); Hadani, Doh & Scheider (2018); Sikavica, Perrault & Rehbein (2020); Goranova & Ryan (2014) | AFL-CIO; IUE-CWA; United Food and Commercial Workers; Utility Workers Union of America. |
| **Non-SRI Activists** | | |
| Corporate | Sikavica, Perrault & Rehbein (2020) | Amalgamated Bank; Harrington Investments, Inc.; Qube Investment Management Inc |
| Hedge & mutual funds | Ryan & Schneider (2002); Goranova & Ryan (2014); Acharya, Gras & Krause (2021). | Clean Yield Asset Management; Azzad Asset Management; GAMCO Asset Management Inc.; Blue Lion Opportunity Master Fund; Voce Capital Management. |
| Public & private pension funds | Guercio & Hawkins (1999); Ryan & Schneider (2002); Goranova & Ryan (2014); Guercio & Woidtke (2019) | California Public Employees' Retirement System; City of Philadelphia Public Employees Retirement System; Massachusetts Pension Reserves Investment Management |
| Other shareholder groups |  | Christopher Reynolds Foundation; David and Annette Jorgensen Revocable Trust; Friends Fiduciary Corporation; National Center for Public Policy Research; The Nathan Cummings Foundation. |

| **Category** | **Authors’ classification** | **Examples (name of shareholder activists)** | |
| --- | --- | --- | --- |
|  |  | **SRI Activist** | **Non-SRI Activist** |
| Individual shareholders | We retrieved the names of all individual shareholders (i.e., private persons) who sponsored more than five proposals during the analysis period. These 32 individuals out of 288 (11%) sponsored 89% of all the proposals sponsored by the individual shareholder category in our sample. Using Google searches and LinkedIn, we identified “*what they do and how they describe who they are*” (Sikavica et al., 2020:1238) to classify them as SRI or non-SRI. | Zhao, Jing; Harrington, John C.; Cohen, Jack; Strobhar, Thomas; Ridenour, David. | Chevedden, John; Steiner, Kenneth; McRitchie, James; Steiner, William; Young, Myra. |
| Coordinated | This category comprises proposals that are cosponsored by more than one shareholder activist, and many proposals are cosponsored by shareholder activists of different types. We classified these proposals as sponsored by SRI activists if at least one of the shareholder activists is an SRI, according to our classification presented above. The remaining cosponsored proposals, which are sponsored only by non-SRI activists, were tagged as non-SRI.  Bauer, Moer & Viehs (2015) | Benedictine Sisters of Baltimore\|\|Benedictine Sisters of Monasterio Pan de Vida\|\|Folksam Group\|\|Franciscan Sisters of Perpetual Adoration\|\|Providence Trust\|\|Sisters of the Humility of Mary;  Brian Patrick Kariger Revocable Trust\|\|Dobson, Andrew P.\|\|Mother Earth Foundation\|\|K.F.P. A California Limited Partnership\|\|The Shared Earth Foundation;  Benedictine Sisters of Cullman, Alabama, Inc.\|\|Benedictine Sisters of Mount St. Scholastica\|\|Brian Patrick Kariger Revocable Trust\|\|K.F.P. A California Limited Partnership\|\|Mercy Investment Services\|\|Presbyterian Church USA;  Amalgamated Bank\|\|Benedictine Sisters of Mount St. Scholastica\|\|Green Century Capital Management\|\|Impax Asset Management | Amalgamated Bank\|\|Amalgamated Bank LongView LargeCap 500 Index Fund\|\|Broad Market 3000 Fund  Mayberry LLC\|\|Persephone LLC\|\|The Sundance Family Foundation\|\|Trillium P21 Global Equity Fund |

**Table A3.** List of keywords used to classify shareholder proposals’ expected outcomes

From our sample of 6,446 shareholder-initiated proposals covering the period of 2013 to 2021, we identified 1,801 unique syntax of proposal descriptions. We broke down these unique descriptions using natural language processing to build our list or key words, which were further related to SOSA and non-SOSA categories, as shown in the table below.

| ***Category*** | ***Keywords SOSA proposals*** |
| --- | --- |
| Community  (C) | **“community” +** < charitable contributions; abortion; anti-traditional family; cannabis; economic inequality; educate; effect; exposure; homosexual lifestyle; homosexuality; humanitarian; impact; low income; marriage definition; minority; plant closure + impact; pride flag; pro discrimination law; public harm; public health; public harm; side effects; levaquin victims; uninsured; boy scout america > |
| Diversity  (D) | **“employee” +** <diversity; diversity + eeo; diversity + eeoc; diversity + female percentile; diversity + gays; diversity + lgbt; diversity workplace; diversity+ management; ethnicity gap; gender + race + ethnicity + gap; gender gap; management + diversity; race + ethnicity + gap; racial + gender + diversity; executive + diversity > |
| Employee  (ER) | **“employee” +** < disparity + pay; ability; accident; antidiscrimination; background check; claim; code; code + truth; code of conduct; compensation + redistribution; complain; compliance eeoc; cost food; costofliving; coversion reit; development; discrimination; discrimination + gender; discrimination + macbride; discrimination + sexual orientation; eeo; eeo + gender; eeo + sexual orientation; eeo policy; eligibility; employement + opportunity; employment; ethical + labor; fairfoodprogram; family leave; gender + eeo; gender + inequity; gender + racial; gender paygap; health care; holy land principle; human capital; ilo code conduct; ilo labor; incentive; incidents + worker; inequality; inequitable; job outsource; labor discrimination; litigation; minimum wage; nondiscrimination + policy; ombudsman; pay disparity; pay gap; paygap; pension + underfunded; pension plan; political retribution; prison labor + compliance; prison labor + supply; prison labor + usage; reimbursement; retirement plan; safety; safety + compliance; savings + job act; sexual harassment; sexual orientation; sexual orientation antibias; smoke area; time off; unemployment + inequality; vote + benefits; work condition; work practices + safety; worker practices; worker pay; worker wage + hour; workplace safety; annuity contract; gender equality; discrimination + pregnant; inequitable; gender + race ethnicity + equity > |
| Human rights  (HR) | **“human rights” +** <antiimmigration; burma; child + exploitation; child + labor; sexual + exploitation; china; chinese; civil assessment; civil rights; code conduct; committee + policy; country + selection; due diligence; forced labor; freedom expression; genociderelated; high risk; immigrant; impact; indicator; indigenous; policy; recidivism; religious freedom; reproductive; slavery; supply chain; trade partnership; traffic + labor; social + holy land principle > |
| Product  (P) | **“customer” +** < overdraft practices; seat size; service; privacy security; data privacy; data security; customer + optout; customer satisfaction; data + customer recognition; consumer data >  **“product” +** < 5g; access; accessibility; accidents; advertising; advertising principle; affordability; antibiotic + disease; antibiotic + supply chain; antibiotic + use; antiforced labor + supply chain; autodial; big data; bpa + use; care testing; chemical + principle; cloudbased; content + web; country selection; crime; drug + lobby; drug distribution + drug price; facial recognition; fair lending; firearm; footprint + porfolios; product + fsccertified; genetic engineering; genetically engineer; genetically modify; gmo + ingredients; gmo + use; gun; hate speech; health impact; internet + principle; labeling + gmo; lables; lending compliance; made usa; mercury; mercury amalgam; nanomaterials; neonics; neutrality principle; nutrition goal; obesity; obesity + fast food; open access; opioid; overdraft; patent file; pesticide + use; pharmaceutical price; predatory lend; principle care; prison; protein; reboot; release; responsibility; responsible + supply chain; responsible suppliers; responsible supply; safety + maintenance; safety + virus control; sandy hook principle; sell gun; sfatey; smokefree; smoking + tobacco; sourcing materials; stem cell; product + sugar; product + sugar + supply chain; product + supplier + code conduct; product + supplier + code of practice; product + supply chain; sustainable + purchasing; takeback policy; toxicity; truthful news; unsafe; violence; weapon; youth + marketing; safety >  **“product + animal” +** < abuse; cage; cagefree; cattle dehorn; cruelty; dehorn; experiment; feather; fur; gestation; laboratory; testing; wefare; wild >  **“product + tobacco” +** < advertising; marketing; tobaccorelated; sale > |
| Emissions  (GHG) | **“emissions” +** < ghg; air; co2; methane; carbon; carbon dioxide goal; carbon economy; carbon footprint; carbon tax; footprint + porfolios; ghg goal; zero + ghg; fossil fuel; fuel; goal; netzero; goal + scenario; goal + science; sciencebased; paris goal; product operation; greenhouse; greenhouse gas >  **“climate” +**<action; change; goal; opportunity; principle >  **“coal” +** <combustion; degree scenario; fossil fuel; eeo; emission + tri; ash; use; reliance>  **“energy” +** <efficiency; renewable; wind turbine; goal; management; nuclear + facility; nuclear power; solar energy >  **“environment” +** < lowcarbon; meet degree scenario; methane; netzero; oil sand pollution; oilwells; paris agreement; paris goal; pollution + hog production; sciencebased + goal; warming; warmingrelated; 2degree; biomass; two degree scenario > |
| Waste mgmt.  (WM) | **“waste” +** < coal; food; goal; nuclear + safety; nuclear; oil recovery; packaging; radioactive >  **“plastic”** + < bag; pollution; straw >  **“recycle” or “recycling” +** < beverage; packaging; waste; foam packaging; hazard; impact + foam cup; nonrecyclable > |
| Water  (W) | **“water” +** < usage; use; impact; seacanal; supply chain; access; supply; pollution; quality; waterrelated; stormwater; hydraulic fracture; hydraulic fracturing; fracking; toxic fracking > |
| Environment miscellaneous.  (EM) | **“environment” or “environmental” +** coast wetland; crude oil + natural gas; deforestation; deforestation + supply chain; tar sand; impact; climate + supply chain; mining + mountaintop; mountaintop operation; natural gas + carbon price scenario; nuclear + safety; opportunity + climate; palm oil; rainforest; wood; climate lobbying; counterproposal; duediligence; fracturing; goal; harm; impact; impact + mining; impact + solar; impact + wind; mining; mitigation; nuclear safety; palm oil; remediation; environment related; mining + impact + financing > |
| Sustainability disclosure  (SD) | **“disclose” or “disclosure” +** scenario analysis; sustainable development goals; sdg; climate financing; eeo data; esg; esg + metrics; climate change; ghg; paris alignment >  **“report” +** climate lobbying; cyber risk; carbon principle; climate; climate risk; climaterelated; controversy; E&S + ghgs; emission + paris goal; esg performance; ghg + water; ghg emission; greenhouse emission; physical risk + climate; political analysis; political contributions; purpose corporation >  **“report + risk assessment” +** human rights; conflict areas; climate change; social issues; hazardous materials; oil sand; sustainability; water concern; water risks >  **“sustainability” +** < plan; reporting; report; sasb; gri > |

| ***Category*** | ***Keywords non-SOSA proposals*** |
| --- | --- |
| Auditor related  (AUD) | **“audit” or “auditor” +** < compliance; every two year; external + business plan; report + internal control; rotation; selection; appointment; independence; report; rotate > |
| Business operations  (BO) | **“ operation” +** < investment; logistics; market potential; markets; plant closure; price stability; manufacture site selection; Replacement; impact + california fire; communication agency; value + company sale; value + cross selling; value + non-core operations; sales + business > |
| Board of directors  (BOD) | **“board” +** < advisory position; attendance; audit committee; chairman; conflict; declassify; diversity; diversity + selection; employee representation; employment + neos; esg oversight; female; fiduciary obligation; human capital + experience; human rights + experience; independent; independent chair; independent + chairman; independent lead director; matrix; meeting; nomination + policy; nonmanagement + director; number; other companies; oversight; oversight + privacy security; political contribution; political goal; qualifications; separate position + chair; size + approval; supermajority + decision; task >  **“board committee” +** < climate oversight; stockholder value; climate; csr; environmental; human rights; international policy; public policy; risk management; societal risk; special; sustainability >  **“director” +** < board memberships; climate + experience; committee + membership; compensation; compensation + non-employee; duties; duty; election; election + majority vote; environmental + experience; environmental + expertise; experience; expertise; expertise + civil rights; indemnification; lead + qualification; metric + experience; nomination; nomination + open seat; nomination + policy; pay; qualification + experience; qualification matrix; removal; removal + policy; retention; retirement + age ; selection; stock ownership + policy; term limit; committee membership; election; experience + human rights; multiple boards; remove; tenure > |
| Codes, policy, standards  (CPS) | **“article” or “bylaw” or “charter” +** < amend; amend articles; Amendment; shareholder approval; antitakeover provision; approval; charter; Delaware; ownership threshold; precatory; record date; shareholder right; shareholder provision; venue provision; written consent; dakota + reincorporate; delaware + incorporate; insider selling; jersey + reincorporate; stockholder litigation > |
| Executive compensation  (EXC) | **“compensation” or “incentive” +** < ceo + annual exam; ceo + clawback + payment; ceo + ceo retirement; adjustment + executive; benchmarking; benchmarking + ceo bonus; bonus banking + executive; buyback + executive; carbon emission + executive; cash bonus + executive; cash incentive + executive; ceo + cfo; ceo + payratio; changeincontrol + executive; clawback + executive; clawback payment + ceo; clawback policy; committee + ceo; compliance cost; consultant; death benefits + executive; disclosure + executive; diversity + performance metric + executive; employment + other companies; employment + other companies + board seat + ceo; equity + change in control; equity + performance metric; equity + vesting; equity award + executive; equity award + vesting + executive; equity plan; equity retirement + executive; esg + executive; executive + hedge stock; executive + share buyback; executive + stockbased; expert + executive; gaap + executive; gaap merics + executive; golden coffin; golden parachute; grant + ceo; human rights + metrics + executive; plan; incentivebased; legal compliance + cost; legal cost + executive; limit + executive; metric + ceo; metric + esg; metric + executive; metric + ghg + executive; miscellaneous + executive; named executive officers; nondeductible + executive; package; payforperformance + executive; payratio + executive offices; pension + executive; performancebased + equity; perquisites + executive; poison pill; policy + executive; principle + executive; report; roi + executive; sayonpay + executive; sell stock + executive; serp; severance agreement; severance reward + ceo; share repurchase + executive; share retention + executive; shares + vesting; social criteria + executive; stock + bonus + executive; stock award + executive; stock option; stock retention + executive; stockbased; sustainability + executive; sustainability performance + executive; vested + awards; vesting + accelerate; vestion + options; metric + retirement plan; disclosure + senior officer; equity + stock awards; equity awards + fair value; penalty fine > |
| Financing  (FIN) | **“equity” +** <capital; capital repurchase; issue stock; plan + capitalization; recapitalization + preferred stock; share buyback; share reissuances; spinoff; stock + conversion; stock + new shares; stock buyback; stock class; stock retention; stock split; transaction; vesting; changeincontrol >  **“finance” or “financing” +** < analysis + asset; capital expenditure; capital transaction; cost + investment; cost and benefits; debt; equity + trading; equity related; financing + operations; funding; hedge transaction; profit >  **“value” +** < disinvestment; tax saving; collateral trade; hedge + transaction; holding company + drawback; loan; shareholder > |
| Governance  (G) | **“adopt” or “implement” or “policy” +** < alternatives + maximize shareolder value; business standards; campaign + free speech; corporate governance + guidelines; corporate values; credit rating; denial + construction; disallow selfscheduling; ethic + code; ethical contract; fraud; executive + retention; executive + succession planning; foreign sale; material risk; moral hazard; payment + government; payment judiciary; performance metrics; performance standards; purpose corporation; regulator + share counterpart; return capital + shareholder; risk management; shareholder + equity retention; sox + stress; strand asset; strategy + sale asset; tax strategy >  **“ceo” +** < replace; resign; resignation; retention; succession planning; code of ethic; contractual + provision >  **“governance” +** < compliance; lobbying organization; miscellaneous; policy; related + specific; risk management; cybersecurity incident; whistleblower; government + payment; government + subsidy; investor claim + roi; lawsuit; liability; liability + discharge >  **“lobby” or “lobbying” +** < payment; federal level; political expense; political >  **“meeting” +** < call; special; shareholders; annual + in-person; date time; inperson; ownership threshold; rotate location; scheme arrangement; special purpose; voting + shareholding threshold >  **“political” +** < alec + membership; contribution; spending; electoral; activities; contributions + disclosure; electioneering; influence + election; lobby payment; nonpartisanship; spending + election; disclosure; expenditure; spending >  **“shareholder” +** < change control; poison pill + rights; policy; rights; rights + proposals supported; stock retention; value + acquisition; value + cash flow + return shareholder; divest; divest + segment; financial flow >  **“strategy” +** < shareholder value + maximize; value + maximize; shareholder value + optimize; company values + political activity; corporate values; risk management + cybersecurity incident; severance agreement + changeincontrol > |
| Income allocation  (INC) | **“dividend” +** < share buyback; dividend payment; financial; payment; payout; share buybacks over dividend; loan repayment; income distribution >  **“value” +** < acquisition + shareholder value; aquired entity; repay; tax saving > |
| Transparency  (TR) | **“disclose” or “disclosure” or “report” +** < annual report; government payment; plan; purpose corporation; corporate purpose; taxexemption; debt; derivatives; electronically information >  **“report” +** < risk assessment + investment; tax principle; withdrawn proposals >  **“transparency” +** < public policy; fake news; tax principle; policies; annual report; government information; government service; restatements; withdrawn resolution > |
| Voting rights  (VOTE) | **“equity” +** < dual stock; equity + dualclass stock; dualclassstock; onevote per share; shareholder + onevote per share; ownership + holding requirements >  **“proxy” +** < access; acess + right; advisor; contest cost; reform; voting; voting + disclosure; tabulation; voting + tabulation; confidentiality >  **“vote” +** < abstentions; confidential; count; counting methodology; cumulative; onevotepershare; ownership threshold; shareholder + notice; shareholder right; shareholder + voting rights; shareholder approval; simple majority; supermajority; voting + policy; voting + rights > |

**Table A4.** Illustrative Coding and Measurement of Shareholder Proposal Activism Heterogeneity

| *Panel A - Amazon 2020’s distribution of proposals by activist identity vs. proposals’ type (N = 20)*  ***[between brackets = A, B, C, or D quadrant]*** | | | | |
| --- | --- | --- | --- | --- |
|  |  | **Proposal type** | |  |
|  |  | Non-SOSA | SOSA | **Total** |
| **Activist identity** | Non-SRI | 2 **[A]** | 8 **[B]**  (Soc.=3, Env. =0) | 10 |
|  | SRI | 1 **[C]** | 9 **[D]**  (Soc.=6, Env. =0) | 10 |
|  | **Total** | 3 | 17 | 20 |

| *Panel B – Example of calculation of Heterogeneity indices* | | | |
| --- | --- | --- | --- |
| **Label** | **Description** | **Quadrants** | **Index** |
| **Identity-Proposal Het.** *(former AH-I)* | Heterogeneity of shareholder proposal activism combining both attributes: the identity (SRI and non-SRI) of shareholder activists sponsoring the proposals and the type of demand (SOSA and non-SOSA) of these proposals. | ***A, B, C, D*** | $1 -[\left( \frac{2 \left[ A \right]}{20 \left[ ABCD \right]} \right)^{2}+ \left( \frac{8 \left[ B \right]}{20 \left[ ABCD \right]} \right)^{2}{+\left( \frac{1 \left[ C \right]}{20 \left[ ABCD \right]} \right)}^{2}+\left( \frac{9 \left[ D \right]}{20 \left[ ABCD \right]} \right)^{2}=0.625$ |
| **Identity Het if SOSA** | Heterogeneity in the identity of shareholder activists (SRI vs non-SRI) sponsoring proposals classified as socially oriented (SOSA). | ***B, D*** | $1 -\left[ \left( \frac{8 \left[ B \right]}{17 \left[ B+D \right]} \right)^{2}+ \left( \frac{9 \left[ D \right]}{17 \left[ B+D \right]} \right)^{2} \right]=0.498$ |
| **Proposal Het by non-SRI** | Heterogeneity in the type of demands (SOSA vs non-SOSA) within shareholder-initiated proposals sponsored by activists that are not identified as socially responsible investors (non-SRI). | ***A, B*** | $1 -\left[ \left( \frac{2 \left[ A \right]}{10 \left[ A+B \right]} \right)^{2}+ \left( \frac{8 \left[ B \right]}{10 \left[ A+B \right]} \right)^{2} \right]=0.320$ |
| **Diagonal Het.** | Heterogeneity based only on the groups of proposals for which the activist’s identity conflicts with the type of proposal’s demand; using the proportion of proposals sponsored by SRI activists putting forward a non-SOSA demand, and the proportion of proposals sponsored by non-SRI activists putting forward a SOSA demand. | ***B, C*** | $1 -\left[ \left( \frac{8 \left[ B \right]}{9 \left[ B+C \right]} \right)^{2}+ \left( \frac{1 \left[ C \right]}{9 \left[ B+C \right]} \right)^{2} \right]=0.198$ |

**Table A5.** Regression results for ESG Decoupling on Proposal Heterogeneity, full sample, with a correction for sample selection

|  | **Dependent variable: E&S Decoupling** | | | | **Dependent variable: ESG Decoupling** | | | |
| --- | --- | --- | --- | --- | --- | --- | --- | --- |
|  | **1** | **2** | **3** | **4** | **5** | **6** | **7** | **8** |
| Target=1 # Identity-Proposal Het. (lag) | 2.57 |  |  |  | 2.88^*^ |  |  |  |
|  | [1.34] |  |  |  | [1.82] |  |  |  |
| Target=1 # Identity Het. if SOSA (lag) |  | 4.55 |  |  |  | 5.80^**^ |  |  |
|  |  | [1.47] |  |  |  | [2.34] |  |  |
| Target=1 # Proposal Het. by Non-SRI (lag) |  |  | 4.70^*^ |  |  |  | 5.85^**^ |  |
|  |  |  | [1.65] |  |  |  | [2.51] |  |
| Target=1 # Diagonal Het. (lag) |  |  |  | 4.07^*^ |  |  |  | 4.65^***^ |
|  |  |  |  | [1.91] |  |  |  | [2.61] |
| Inst. Ownership (lag) | -5.27^*^ | -5.21^*^ | -5.23^*^ | -5.26^*^ | 0.57 | 0.63 | 0.61 | 0.58 |
|  | [-1.86] | [-1.84] | [-1.84] | [-1.85] | [0.25] | [0.28] | [0.27] | [0.26] |
| Entrenchment (lag) | 0.21 | 0.21 | 0.21 | 0.22 | 0.43 | 0.43 | 0.43 | 0.43 |
|  | [0.50] | [0.50] | [0.50] | [0.51] | [1.32] | [1.32] | [1.31] | [1.33] |
| Size (lag) | 1.69^***^ | 1.70^***^ | 1.70^***^ | 1.70^***^ | 0.09 | 0.09 | 0.09 | 0.09 |
|  | [5.64] | [5.67] | [5.67] | [5.67] | [0.35] | [0.38] | [0.38] | [0.39] |
| ROA (lag) | -0.72 | -0.83 | -0.75 | -0.77 | -0.49 | -0.64 | -0.53 | -0.55 |
|  | [-0.18] | [-0.20] | [-0.18] | [-0.19] | [-0.15] | [-0.20] | [-0.17] | [-0.17] |
| Investment (lag) | -9.98 | -9.93 | -9.98 | -9.70 | -9.12 | -9.02 | -9.10 | -8.79 |
|  | [-1.07] | [-1.06] | [-1.07] | [-1.04] | [-1.24] | [-1.23] | [-1.25] | [-1.20] |
| Org. Slack (lag) | 4.22^*^ | 4.25^*^ | 4.22^*^ | 4.23^*^ | 0.49 | 0.52 | 0.49 | 0.49 |
|  | [1.74] | [1.75] | [1.74] | [1.74] | [0.26] | [0.28] | [0.26] | [0.27] |
| Target=1 # Inst. Ownership (lag) | -7.82^**^ | -7.87^**^ | -7.97^**^ | -8.02^**^ | -7.46^**^ | -7.36^**^ | -7.51^**^ | -7.66^**^ |
|  | [-1.98] | [-1.99] | [-2.01] | [-2.02] | [-2.43] | [-2.43] | [-2.44] | [-2.49] |
| Target=1 # Entrenchment (lag) | -0.73 | -0.72 | -0.70 | -0.74 | -0.28 | -0.27 | -0.25 | -0.29 |
|  | [-0.98] | [-0.98] | [-0.95] | [-1.00] | [-0.49] | [-0.47] | [-0.43] | [-0.51] |
| Target=1 # Size (lag) | 0.91^**^ | 0.94^***^ | 0.92^**^ | 0.93^**^ | 0.58^**^ | 0.59^**^ | 0.57^*^ | 0.59^**^ |
|  | [2.50] | [2.67] | [2.49] | [2.57] | [1.98] | [2.08] | [1.92] | [2.03] |
| Target=1 # ROA (lag) | 9.26 | 9.22 | 9.64 | 9.63 | 6.34 | 6.27 | 6.81 | 6.76 |
|  | [1.32] | [1.32] | [1.38] | [1.38] | [1.11] | [1.10] | [1.19] | [1.19] |
| Target=1 # Investment (lag) | 18.85 | 18.42 | 19.54 | 18.45 | 15.35 | 14.66 | 16.09 | 14.87 |
|  | [1.19] | [1.15] | [1.24] | [1.16] | [1.32] | [1.24] | [1.40] | [1.27] |
| Target=1 # Org. Slack (lag) | -2.59 | -2.50 | -2.34 | -2.63 | 2.27 | 2.34 | 2.55 | 2.22 |
|  | [-0.53] | [-0.51] | [-0.47] | [-0.54] | [0.58] | [0.61] | [0.65] | [0.57] |
| Target=1 # Withdraw/Total (lag) | 1.20 | 1.05 | 1.11 | 1.17 | 1.57^*^ | 1.41 | 1.49 | 1.54^*^ |
|  | [1.02] | [0.89] | [0.94] | [0.99] | [1.74] | [1.55] | [1.64] | [1.70] |
| Target=1 # Omitted/Total (lag) | 1.10 | 1.02 | 1.02 | 1.09 | 0.46 | 0.40 | 0.39 | 0.46 |
|  | [0.86] | [0.80] | [0.80] | [0.86] | [0.49] | [0.42] | [0.41] | [0.48] |
| Constant | -23.15^***^ | -23.33^***^ | -23.20^***^ | -23.24^***^ | 1.09 | 0.89 | 1.06 | 0.99 |
|  | [-5.46] | [-5.53] | [-5.50] | [-5.49] | [0.31] | [0.25] | [0.30] | [0.28] |
| R2-Adjusted | 0.241 | 0.241 | 0.241 | 0.241 | 0.225 | 0.226 | 0.226 | 0.226 |
| Observations | 5577 | 5577 | 5577 | 5577 | 5577 | 5577 | 5577 | 5577 |

**Notes:** see variables definition at the bottom of Table 3**.** * *p* < 0.1, ** *p* < 0.05, *** *p* < 0.01. Standard errors clustered by firm. Year fixed effects and industry fixed effects included.

**Table A6.** Robustness tests, Regression results for ESG Decoupling on Shareholder Proposals Activism Heterogeneity,

standard errors clustered by firm *(Control variables included but untabulated)*

|  | **E&S Decoupling** | | | | **ESG Decoupling** | | | |
| --- | --- | --- | --- | --- | --- | --- | --- | --- |
| **Independent variable =** | Identity-Proposal Het. (lag) | Identity Het. if SOSA (lag) | Proposal Het. by non-SRI (lag) | Diagonal Het. (lag) | Identity -Proposal Het. (lag) | Identity Het. if SOSA (lag) | Proposal Het. by non-SRI (lag) | Diagonal Het. (lag) |
| **Panel A – Entropy balanced sample** | | | | | | | | |
|  | 9.23^***^ | 10.21^***^ | 9.90^***^ | 10.21^***^ | 7.71^***^ | 10.26^***^ | 9.39^***^ | 8.87^***^ |
|  | [4.08] | [3.05] | [3.34] | [4.18] | [3.81] | [3.69] | [3.72] | [4.15] |
| R2-Adjusted | 0.349 | 0.343 | 0.344 | 0.347 | 0.304 | 0.301 | 0.301 | 0.303 |
| Observations | 3457 | 3457 | 3457 | 3457 | 3457 | 3457 | 3457 | 3457 |
| **Panel B – Proposals heterogeneity (Activism) three years average** | | | | | | | | |
|  | 12.65^***^ | 18.77^***^ | 18.18^***^ | 17.29^***^ | 10.75^***^ | 18.40^***^ | 17.90^***^ | 15.60^***^ |
|  | [3.30] | [2.80] | [2.84] | [3.32] | [3.36] | [3.48] | [3.39] | [3.61] |
| R2-Adjusted | 0.206 | 0.203 | 0.204 | 0.206 | 0.166 | 0.166 | 0.168 | 0.168 |
| Observations | 2528 | 2528 | 2528 | 2528 | 2528 | 2528 | 2528 | 2528 |
| **Panel C - Excluding sample firms targeted with more than five proposals in a given year** | | | | | | | | |
|  | 4.77^**^ | 7.02^*^ | 6.90^**^ | 6.55^***^ | 3.55^**^ | 6.08^**^ | 6.16^***^ | 5.19^***^ |
|  | [2.40] | [1.96] | [2.43] | [2.90] | [2.11] | [2.16] | [2.61] | [2.76] |
| R2-Adjusted | 0.252 | 0.252 | 0.252 | 0.253 | 0.206 | 0.206 | 0.207 | 0.207 |
| Observations | 3293 | 3293 | 3293 | 3293 | 3293 | 3293 | 3293 | 3293 |
| **Panel** **D** **- Excluding the top 5% of targeted firms in terms of activism of heterogeneity** | | | | | | | | |
|  | 5.72^***^ | 10.06^*^ | 19.30^*^ | 9.67^***^ | 4.59^**^ | 10.59^**^ | 23.58^**^ | 9.27^***^ |
|  | [2.69] | [1.96] | [1.65] | [2.93] | [2.59] | [2.53] | [2.24] | [3.35] |
| R2-Adjusted | 0.253 | 0.260 | 0.257 | 0.261 | 0.206 | 0.209 | 0.210 | 0.212 |
| Observations | 3320 | 3295 | 3117 | 3170 | 3320 | 3295 | 3117 | 3170 |
| **Panel E - Excluding the bottom 5% of targeted firms in terms of ESG performance** | | | | | | | | |
|  | 5.44^***^ | 7.89^**^ | 8.35^***^ | 6.99^***^ | 4.73^***^ | 8.20^***^ | 8.01^***^ | 6.31^***^ |
|  | [2.71] | [2.39] | [2.79] | [3.07] | [2.75] | [3.06] | [3.19] | [3.26] |
| R2-Adjusted | 0.268 | 0.267 | 0.268 | 0.268 | 0.214 | 0.214 | 0.215 | 0.214 |
| Observations | 3308 | 3308 | 3308 | 3308 | 3308 | 3308 | 3308 | 3308 |

|  | **E&S Decoupling** | | | | **ESG Decoupling** | | | |
| --- | --- | --- | --- | --- | --- | --- | --- | --- |
| **Independent variable =** | Identity-Proposal Het. (lag) | Identity Het. if SOSA (lag) | Proposal Het. by non-SRI (lag) | Diagonal Het. (lag) | Identity -Proposal Het. (lag) | Identity Het. if SOSA (lag) | Proposal Het. by non-SRI (lag) | Diagonal Het. (lag) |
| **Panel F – Industry adjusted (Activism) proposals heterogeneity** | | | | | | | | |
|  | 5.63^***^ | 8.26^**^ | 8.25^***^ | 7.11^***^ | 4.81^***^ | 8.23^***^ | 8.10^***^ | 6.46^***^ |
|  | [2.86] | [2.53] | [2.85] | [3.22] | [2.87] | [3.11] | [3.33] | [3.45] |
| R2-Adjusted | 0.263 | 0.262 | 0.263 | 0.263 | 0.213 | 0.214 | 0.215 | 0.214 |
| Observations | 3457 | 3457 | 3457 | 3457 | 3457 | 3457 | 3457 | 3457 |
| **Panel G - Replacing industry FE by a dummy marking E&S sensitive industry** | | | | | | | | |
|  | 3.70^*^ | 6.07^*^ | 5.29^*^ | 4.93^**^ | 4.01^**^ | 7.42^***^ | 6.89^***^ | 5.64^***^ |
|  | [1.94] | [1.88] | [1.85] | [2.29] | [2.54] | [2.86] | [2.90] | [3.13] |
| R2-Adjusted | 0.223 | 0.223 | 0.223 | 0.223 | 0.189 | 0.190 | 0.190 | 0.189 |
| Observations | 3457 | 3457 | 3457 | 3457 | 3457 | 3457 | 3457 | 3457 |
| **Panel H – Decoupling using ESG performance scores two years ahead** | | | | | | | | |
|  | 4.86^**^ | 7.19^**^ | 8.84^***^ | 5.45^**^ | 4.35^**^ | 7.47^**^ | 8.53^***^ | 5.39^***^ |
|  | [2.20] | [1.97] | [2.84] | [2.17] | [2.35] | [2.56] | [3.34] | [2.61] |
| R2-Adjusted | 0.248 | 0.248 | 0.250 | 0.248 | 0.170 | 0.170 | 0.173 | 0.170 |
| Observations | 2918 | 2918 | 2918 | 2918 | 2918 | 2918 | 2918 | 2918 |
| **Panel I – Decoupling calculated with a three-year moving average of performance score & Firm Fixed Effects** | | | | | | | | |
|  | 3.20^**^ | 2.11 | 5.81^***^ | 3.77^**^ | 2.11^*^ | 1.55 | 4.41^***^ | 2.56^*^ |
|  | [2.01] | [0.86] | [2.80] | [2.03] | [1.84] | [0.87] | [2.95] | [1.92] |
| R2-Adjusted | 0.190 | 0.189 | 0.192 | 0.190 | 0.181 | 0.180 | 0.184 | 0.182 |
| Observations | 2487 | 2487 | 2487 | 2487 | 2487 | 2487 | 2487 | 2487 |
| **Panel J - Activism heterogeneity calculated with only proposal sponsored by institutional shareholders** | | | | | | | | |
|  | 4.50^**^ | 6.80^**^ | 9.74^**^ | 5.67^**^ | 3.93^**^ | 7.16^***^ | 9.83^***^ | 5.19^**^ |
|  | [2.12] | [2.14] | [2.47] | [2.23] | [2.22] | [2.83] | [3.43] | [2.57] |
| R2-Adjusted | 0.261 | 0.261 | 0.261 | 0.261 | 0.211 | 0.212 | 0.212 | 0.211 |
| Observations | 3457 | 3457 | 3457 | 3457 | 3457 | 3457 | 3457 | 3457 |
| **Panel K – Only voted on proposals** | | | | | | | | |
|  | 4.90^*^ | 7.10 | 6.11^*^ | 5.79^**^ | 4.98^**^ | 8.53^**^ | 7.17^**^ | 5.79^**^ |
|  | [1.83] | [1.60] | [1.66] | [2.10] | [2.22] | [2.37] | [2.32] | [2.51] |
| R2-Adjusted | 0.240 | 0.239 | 0.239 | 0.240 | 0.180 | 0.181 | 0.180 | 0.180 |
| Observations | 1519 | 1519 | 1519 | 1519 | 1519 | 1519 | 1519 | 1519 |

**Table A7.** Robustness tests of ESG Decoupling Persistence, Entropy Balancing, robust standard errors, and estimated by Sys-GMM

|  | *Identity-Proposal Het.* | | *Identity Het. if SOSA* | | *Proposal Het. by non-SRI* | | *Diagonal Het.* | |
| --- | --- | --- | --- | --- | --- | --- | --- | --- |
|  | **(1)** | **(2)** | **(1)** | **(2)** | **(1)** | **(2)** | **(1)** | **(2)** |
|  | **Het. = 0** | **Het. > 0** | **Het. = 0** | **Het. > 0** | **Het. = 0** | **Het.> 0** | **Het. = 0** | **Het.> 0** |
| *E&S Decoupling* | | | | | | | | |
| E&S Decoupling (lag) | 0.51^***^ | 0.73^***^ | 0.64^***^ | 0.56^***^ | 0.45^***^ | 0.75^***^ | 0.52^***^ | 0.69^***^ |
|  | [3.44] | [7.20] | [9.41] | [5.67] | [4.91] | [10.44] | [3.93] | [7.35] |
| Observations | 1054 | 1433 | 1752 | 735 | 1553 | 934 | 1183 | 1304 |
| AR(1) | -2.97 | -4.78 | -4.21 | -4.30 | -4.38 | -5.57 | -2.93 | -4.87 |
| AR(1): p-value | 0.00 | 0.00 | 0.00 | 0.00 | 0.00 | 0.00 | 0.00 | 0.00 |
| AR(2) | 1.45 | 0.57 | 1.27 | -1.07 | 1.73 | -0.64 | 1.49 | -0.48 |
| AR(2): p-value | 0.15 | 0.57 | 0.20 | 0.29 | 0.08 | 0.52 | 0.14 | 0.63 |
| Hansen | 23.71 | 20.28 | 55.76 | 47.31 | 39.52 | 46.18 | 20.43 | 21.39 |
| Hansen p-value | 0.16 | 0.32 | 0.08 | 0.26 | 0.20 | 0.06 | 0.31 | 0.26 |
| Nr. Instruments | 59.00 | 58.00 | 84.00 | 79.00 | 75.00 | 71.00 | 60.00 | 56.00 |
| Diff. (1) - (2) (t-stat) | -0.21 (-40.04) | | 0.07 (18.87) | | -0.30 (-91.90) | | -0.18 (-38.61) | |
| *ESG Decoupling* | | | | | | | | |
| ESG Decoupling (lag) | 0.54^***^ | 0.78^***^ | 0.68^***^ | 0.70^***^ | 0.54^***^ | 0.80^***^ | 0.59^***^ | 0.73^***^ |
|  | [3.11] | [9.08] | [10.09] | [7.86] | [5.80] | [9.24] | [3.07] | [9.33] |
| Observations | 1054 | 1433 | 1752 | 735 | 1553 | 934 | 1183 | 1304 |
| AR(1) | -3.26 | -5.89 | -3.81 | -4.96 | -3.46 | -5.99 | -3.21 | -6.07 |
| AR(1): p-value | 0.00 | 0.00 | 0.00 | 0.00 | 0.00 | 0.00 | 0.00 | 0.00 |
| AR(2) | 1.58 | 1.03 | 1.21 | -0.33 | 1.56 | -0.09 | 1.67 | 0.34 |
| AR(2): p-value | 0.11 | 0.30 | 0.23 | 0.74 | 0.12 | 0.93 | 0.10 | 0.73 |
| Hansen | 25.80 | 20.67 | 40.54 | 39.53 | 26.55 | 24.64 | 24.32 | 22.13 |
| Hansen p-value | 0.10 | 0.30 | 0.14 | 0.17 | 0.09 | 0.14 | 0.14 | 0.23 |
| Nr. Instruments | 59.00 | 58.00 | 74.00 | 69.00 | 60.00 | 56.00 | 60.00 | 56.00 |
| Diff. (1) - (2) (t-stat) | -0.24 (-41.25) | | -0.02 (-6.43) | | -0.26 (-71.20) | | -0.14 (-23.23) | |

**Table A8.** Mean Covariates Balance

|  | *Panel A: Original Sample* | | | | *Panel B: After Entropy Balancing* | | |
| --- | --- | --- | --- | --- | --- | --- | --- |
|  | **Mean Treated** | **Mean Control** | **Mean difference** | **P-value of difference** | **Mean Control** | **Mean difference** | **P-value of difference** |
|  | **(1)** | **(2)** | **(1)-(2)** | **(1)-(2)** | **(3)** | **(1)-(3)** | **(1)-(3)** |
| Institutional ownership | 0.754 | 0.833 | -0.079 | 0.000 | 0.754 | 0.000 | 1.000 |
| Entrenchment | 3.387 | 3.618 | -0.231 | 0.000 | 3.387 | 0.000 | 1.000 |
| Size | 10.468 | 9.067 | 1.401 | 0.000 | 10.468 | 0.000 | 1.000 |
| ROA | 0.139 | 0.146 | -0.007 | 0.026 | 0.139 | 0.000 | 1.000 |
| Investment | 0.053 | 0.043 | 0.010 | 0.000 | 0.053 | 0.000 | 1.000 |
| Organizational Slack | 0.123 | 0.131 | -0.008 | 0.121 | 0.123 | 0.000 | 1.000 |
| Withdraw/Total | 0.116 | 0.123 | -0.007 | 0.506 | 0.116 | 0.000 | 1.000 |
| Omitted/Total | 0.128 | 0.088 | 0.040 | 0.000 | 0.128 | 0.000 | 1.000 |

**Table A9.** Activism Heterogeneity calculated with SRI and non-SRI Activists classification based on PRI signatories

|  | ESG Dec, avg | | | | E&S Dec, avg | | | |
| --- | --- | --- | --- | --- | --- | --- | --- | --- |
| Identity-Proposal Het. (lag) | 4.18^**^ |  |  |  | 4.69^**^ |  |  |  |
|  | [2.48] |  |  |  | [2.35] |  |  |  |
| Identity Het. if SOSA (lag) |  | 5.41^**^ |  |  |  | 5.35 |  |  |
|  |  | [2.02] |  |  |  | [1.54] |  |  |
| Proposal Het. non-SRI (lag) |  |  | 5.81^***^ |  |  |  | 6.91^***^ |  |
|  |  |  | [2.69] |  |  |  | [2.68] |  |
| Diagonal Het. (lag) |  |  |  | 5.45^***^ |  |  |  | 6.10^***^ |
|  |  |  |  | [2.98] |  |  |  | [2.71] |
| Inst. Ownership (lag) | -5.54^*^ | -5.92^*^ | -5.33^*^ | -5.52^*^ | -10.03^**^ | -10.49^**^ | -9.74^**^ | -10.01^**^ |
|  | [-1.71] | [-1.82] | [-1.66] | [-1.71] | [-2.47] | [-2.58] | [-2.42] | [-2.48] |
| Entrenchment (lag) | -0.12 | -0.13 | -0.11 | -0.10 | -0.49 | -0.49 | -0.47 | -0.46 |
|  | [-0.22] | [-0.23] | [-0.20] | [-0.18] | [-0.70] | [-0.71] | [-0.67] | [-0.67] |
| Ln(AT) (lag) | 0.15 | 0.34 | 0.14 | 0.13 | 2.40^***^ | 2.63^***^ | 2.37^***^ | 2.38^***^ |
|  | [0.39] | [0.91] | [0.38] | [0.35] | [5.28] | [5.93] | [5.26] | [5.30] |
| ROA (lag) | 2.14 | 2.19 | 2.36 | 2.08 | 7.09 | 7.21 | 7.32 | 7.03 |
|  | [0.41] | [0.42] | [0.46] | [0.40] | [1.12] | [1.13] | [1.15] | [1.11] |
| Investment (lag) | 7.31 | 7.86 | 6.61 | 6.74 | 13.79 | 14.44 | 12.90 | 13.16 |
|  | [0.58] | [0.62] | [0.52] | [0.53] | [0.85] | [0.89] | [0.80] | [0.81] |
| Org. Slack (lag) | 3.59 | 3.95 | 3.72 | 3.60 | 6.15 | 6.58 | 6.27 | 6.17 |
|  | [1.10] | [1.22] | [1.14] | [1.11] | [1.46] | [1.56] | [1.49] | [1.46] |
| Withdraw/Total (lag) | 1.44^*^ | 1.45^**^ | 1.51^**^ | 1.49^**^ | 1.78^*^ | 1.79^*^ | 1.86^*^ | 1.84^*^ |
|  | [1.96] | [1.98] | [2.06] | [2.03] | [1.85] | [1.86] | [1.94] | [1.91] |
| Omitted/Total (lag) | 0.43 | 0.54 | 0.44 | 0.45 | 1.60 | 1.73 | 1.62 | 1.63 |
|  | [0.47] | [0.60] | [0.49] | [0.50] | [1.32] | [1.42] | [1.34] | [1.35] |
| Constant | 6.34 | 5.00 | 6.43 | 6.48 | -24.51^***^ | -26.10^***^ | -24.28^***^ | -24.36^***^ |
|  | [1.06] | [0.84] | [1.07] | [1.08] | [-3.40] | [-3.64] | [-3.38] | [-3.37] |
| R^2^-Adjusted | 0.212 | 0.211 | 0.213 | 0.213 | 0.262 | 0.261 | 0.263 | 0.262 |
| N | 3457 | 3457 | 3457 | 3457 | 3457 | 3457 | 3457 | 3457 |

*t* statistics in brackets

^*^ *p* < 0.1, ^**^ *p* < 0.05, ^***^ *p* < 0.01

**Table A10.** Regression results for E&S Decoupling on Heterogeneity of Activists sponsoring Environmental and Social proposals

|  | Env Dec | Soc Dec |
| --- | --- | --- |
| Identity Het. if Env. (lag) | 9.56 |  |
|  | [0.94] |  |
| Identity Het. if Soc. (lag) |  | 11.86^*^ |
|  |  | [1.85] |
| Inst. Ownership (lag) | -1.88 | -11.61^**^ |
|  | [-0.38] | [-2.33] |
| Entrenchment (lag) | 0.40 | -1.15 |
|  | [0.50] | [-1.43] |
| Ln(AT) (lag) | 2.38^***^ | 1.75^***^ |
|  | [3.97] | [3.22] |
| ROA (lag) | 1.93 | 4.18 |
|  | [0.21] | [0.51] |
| Investment (lag) | 19.81 | -0.35 |
|  | [1.02] | [-0.02] |
| Org. Slack (lag) | 10.48^**^ | 11.49^**^ |
|  | [2.02] | [2.12] |
| Withdraw/Total (lag) | 5.17^***^ | 2.64 |
|  | [2.71] | [1.63] |
| Omitted/Total (lag) | -1.99 | 3.46 |
|  | [-0.35] | [1.38] |
| Constant | -21.43^***^ | -22.66^***^ |
|  | [-2.87] | [-2.93] |
| R^2^-Adjusted | 0.174 | 0.270 |
| N | 1642 | 1911 |

*t* statistics in brackets

^*^ *p* < 0.1, ^**^ *p* < 0.05, ^***^ *p* < 0.01

Like our other measures of heterogeneity in shareholder activism, *Identity Het. if Env*. and *Identity Het if Soc* are measures of diversity as variety using the Hirschman–Herfindahl Index (HHI). To avoid a mismatch between the dependent variable (*Decoupling*) and the source of proposals heterogeneity, we contrast different activists' identities (SRI vs non-SRI) using only proposals with demands related to either expected environmental or social outcomes. As such, *Identity Het. if Env*. is based on different activists’ categories who sponsored proposals related to environmental issues (i.e., GHG Emissions, Waste Management, Water Usage, and Environment – Misc); while *Identity Het if Soc.*  is based on social issues (e.g., Community, Diversity, Employee, Human Rights and Product). For greater details see the subcategories of SOSA presented in Table 2 – Panel C of the main manuscript

1. Based on Goranova and Ryan (2014) and Baloria, Klassen and Wiedman (2019) categorization/labels. [↑](#footnote-ref-1)
